# CRISPR-KRISPR: a method to identify on-target and random insertion of donor DNAs and their characterization in knock-in mice

**DOI:** 10.1186/s13059-022-02779-8

**Published:** 2022-10-25

**Authors:** Masayuki Tanaka, Keiko Yokoyama, Hideki Hayashi, Sanae Isaki, Kanae Kitatani, Ting Wang, Hisako Kawata, Hideyuki Matsuzawa, Channabasavaiah B. Gurumurthy, Hiromi Miura, Masato Ohtsuka

**Affiliations:** 1 Support Center for Medical Research and Education, Tokai University, Isehara, Kanagawa 259-1193 Japan; 2 Mouse Genome Engineering Core Facility, University of Nebraska Medical Center, Omaha, NE USA; 3 Genome Editing and Education Center Nebraska (GEEC-Nebraska), College of Medicine, University of Nebraska Medical Center, Omaha, NE USA; 4 Department of Pharmacology and Experimental Neuroscience, College of Medicine, University of Nebraska Medical Center, Omaha, NE USA; 5 Division of Basic Medical Science and Molecular Medicine, School of Medicine, Tokai University, Isehara, Kanagawa 259-1193 Japan; 6 The Institute of Medical Sciences, Tokai University, Isehara, Kanagawa 259-1193 Japan

**Keywords:** CRISPR, CIRCLE-seq, Off target, Knock-in, Random insertion, CRISPR-KRISPR, Donor DNA, Intronic region, Repeat element

## Abstract

CRISPR tools can generate knockout and knock-in animal models easily, but the models can contain off-target genomic lesions or random insertions of donor DNAs. Simpler methods to identify off-target lesions and random insertions, using tail or earpiece DNA, are unavailable. We develop CRISPR-KRISPR (CRISPR-**K**nock-ins and **R**andom **I**nserts **S**earching **PR**otocol), a method to identify both off-target lesions and random insertions. CRISPR-KRISPR uses as little as 3.4 μg of genomic DNA; thus, it can be easily incorporated as an additional step to genotype founder animals for further breeding.

**Supplementary Information:**

The online version contains supplementary material available at 10.1186/s13059-022-02779-8.

## Background

Clustered regularly interspaced short palindromic repeats (CRISPR)/CRISPR-associated protein (Cas) nucleases that create double-stranded breaks (DSBs) at the desired loci have been widely used as tools for generating genome-edited animal models [1, 2]. Introduced DSBs are repaired with one of the following two mechanisms: (a) non-homologues end joining (NHEJ) and (b) homology-directed repair (HDR). The *in*sertion and *del*etion (indel) mutations can be introduced into the target region via the NHEJ system, whereas foreign DNA sequences can be inserted into the target locus (knock-in) via the HDR mechanism [3, 4]. NHEJ is generally more efficient than HDR, but knock-in of foreign DNA sequences is of wide interest to the scientific community. For creating knock-in models, a few strategies using either microinjection of long single-stranded DNA (lssDNA) [5] or double-stranded DNA (dsDNA) cassettes as donors have been developed during the past 3–5 years, termed *Easi-*CRISPR [6], *Combi*-CRISPR [7], 2C-HR CRISPR [8], and SPRINT-CRISPR [9], or by in vitro electroporation [10] or in vivo electroporation termed GONAD [11, 12] and *i*-GONAD [13, 14].

CRISPR genome editing technology has two drawbacks: off-target (OT) cleavages and random insertion (RI) of the donor DNA molecules used for creating knock-in alleles. OT cleavage and RI events are generally rare in mouse models created using CRISPR methods. Even if such unwanted lesions exist in the genome, they can be easily segregated. However, there are no simpler methods to identify OT and RI events. With regards to identifying OT events, it is difficult to distinguish them from de novo mutations [15, 16]. Eliminating OT mutations in CRISPR-based genome editing is still challenging [17]. Various methods have been developed to detect OT cleavage sites. They fall into two broad approaches: (1) in silico biased methods and (2) in vivo/in vitro unbiased methods. In silico-based approaches include alignment-based approaches such as Cas-Offinder [18] and scoring-based approaches such as CHOPCHOP [19]. Examples of in vivo unbiased approaches include GUIDE-seq [20] and DISCOVER-seq [21]. These cell-based genome-wide assays are thought to identify OT effects with better precision because they rely on the use of endogenous DNA repair mechanisms. In vitro unbiased approaches include SITE-Seq [22], Digenome-seq [23], and CIRCLE-seq [24]. These biochemical assays allow the detection and quantification of OT effects by in vitro cleavage of naked genomic DNA with Cas9-gRNA ribonucleoprotein (RNP). Among them, CIRCLE-seq is considered as the most sensitive method for detecting OT sites, which relies on cleaving the circularized DNA with RNP and sequencing the flanking sequence of the cleaved site [25]. In addition, verification of in vivo OTs (VIVO) is a method that combines CIRCLE-seq and target amplicon sequencing to effectively evaluate in vivo OT effects [26].

In addition to OT cleavage, insertion of donor DNA at non-target genomic sites is also a concern among genome edited animals. RIs of both dsDNA and lssDNA donors, including their imprecise insertions at on-target sites, have been reported [27, 28]. Characteristics of RI sites and how RIs occur are not well understood. Another commonly encountered problem with the CRISPR animal genome-engineering method is that it would be practically impossible to breed each of the founders to establish separate lines, especially if there are many founder animals containing the desired on target edits. In such scenarios, it would be ideal to screen the founders for unwanted genomic lesions and exclude the ones containing those for breeding and narrowing down to a few founders that do not contain any OT cleavages or RIs.

**Fig. 1:**
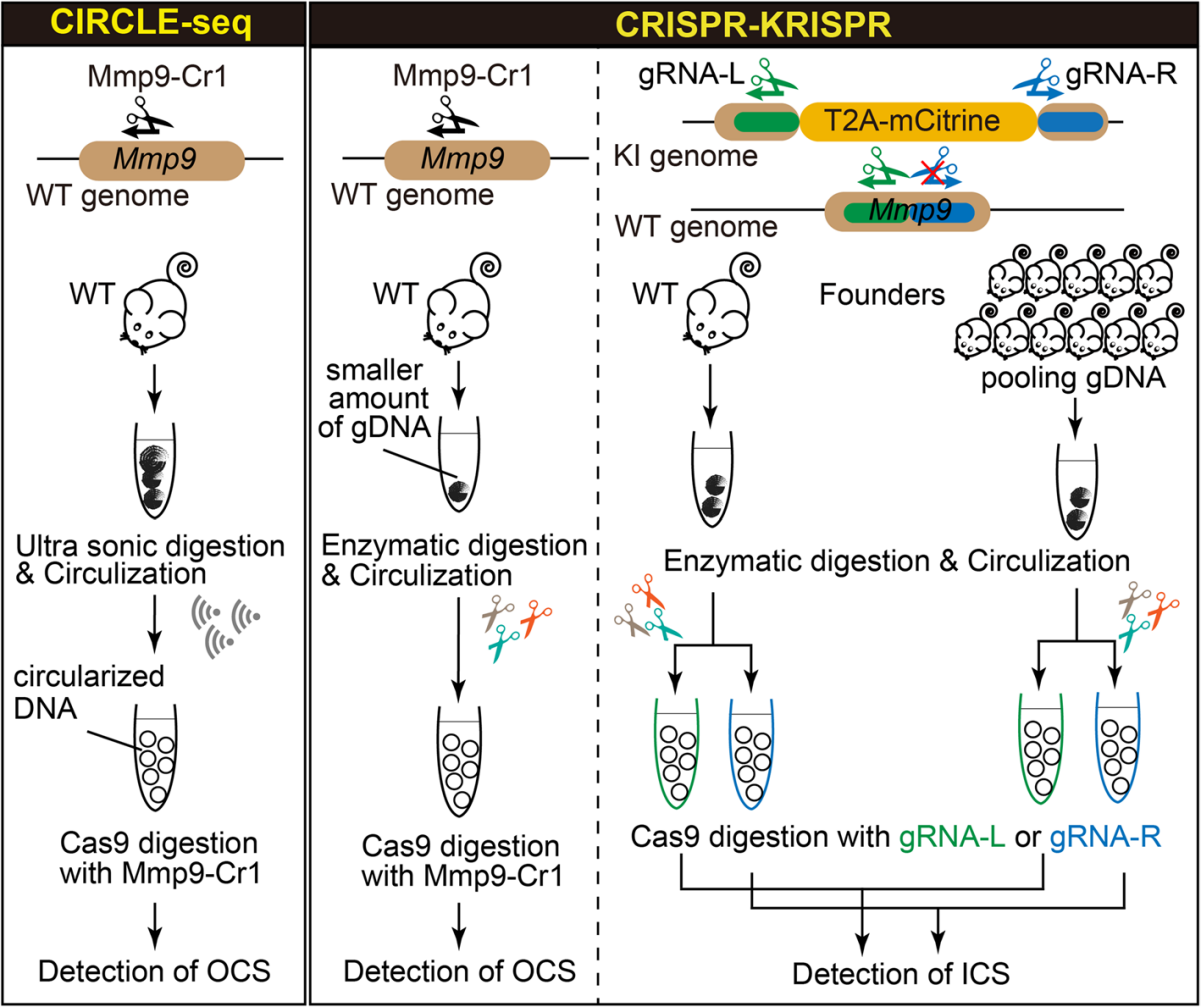
Overview of CIRCLE-seq and CRISPR-KRISPR. Schematic of CIRCLE-seq (left) and CRISPR-KRISPR (right) procedures. The CIRCLE-seq method can identify OT candidate sites (OCSs), whereas CRISPR-KRISPR can identify both OCSs and insertion candidate sites (ICSs)

In this report, we modified the CIRCLE-seq method, which was originally developed for OT cleavages, and developed a method called CRISPR-KRISPR that can identify OT as well as RI events among the mouse models generated using the CRISPR approach (see Fig. 1 for a schematic comparing the CIRCLE-seq and the CRISPR-KRISPR methods). For testing the utility of the CRISPR-KRISPR method, we chose to analyze the entire set of founder mice generated as part of a microinjection-based knock-in experiment to insert a T2A-mCitrine cassette at the mouse *Mmp9* locus [6, 29], with the goal of identifying, and ruling out, OT cleavages and RI events and characterizing them.

## Results

### Modification of the CIRCLE-seq method

We followed the steps to generate circularized DNA, as described in the original CIRCLE-seq method protocol, to obtain 250 ng circularized DNA library [24], but we could only get 12 ng circularized DNA library. This amount of library DNA was not sufficient for downstream processing. We then changed the fragmentation step from sonication (i.e., physical DNA cleavage used in the original CIRCLE-seq protocol) to enzymatic fragmentation to prepare the library (Fig. 1). With this modification, we were able to obtain the required amount of circularized DNA library (>250 ng) from as little as 3.4 μg of gDNA, whereas the original protocol required as much as 25 μg of gDNA (about 7.4 times more). The circularized DNA yield was over 150-fold higher in enzymatic digestion (our modified method) compared to the sonication process used in the original CIRCLE-seq method.

### Modifying the CIRCLE-seq method to identify OT and RI events

Our initial goal was to develop a method to identify OT among the entire set of mice born in a knock-in mouse generation project by using the tail DNA samples. During the process of modifying the CIRCLE-seq method (to identify OT sites), we realized the potential of this method also to identify RIs, by strategically designing the guide RNAs (gRNAs) used in the assay: one gRNA binding to one of the arms and the second one binding to an overlapping region between the insert and the second homology arm. We named this strategy CRISPR-KRISPR (CRISPR- **K**nock-ins and **R**andom **I**nserts **S**earching **PR**otocol)]. One single method (rather than two) to identify both OT and RI would help in identifying the best founder(s), especially if there are many founders containing the targeted insertion of the knock-in cassette.

### A test project to validate the CRISPR-KRISPR method

One of the previously generated knock-in mouse projects (where we inserted a T2A-mCitrine cassette into the *Mmp9* locus [6] (Additional file 1: Fig. S1A)) met this criterion because 67% (8/12) of the founder mice contained the targeted allele, and we wanted to analyze all correctly targeted ones to rule out RIs before choosing the right founder(s) for establishing the knock-in mouse line and to analyze all mice (including non-targeted ones) to comprehensively investigate OTs. Based on the tail DNA genotyping assays using two junction PCRs (one each on 5′ and 3′ junctions), we had identified eight correctly targeted (founders 1, 3, 4, 6, 7, 8, 11, and 12), three untargeted (founders 2, 5, and 9), and two imprecise/partial insertion alleles (founders 4 and 10) (Additional file 1: Fig. S1B). Note that among the correctly targeted animals, one mouse (founder 4) also contained a mosaic imprecise/partial insertion allele [6]). For the CRISPR-KRISPR analysis, we could not include founder 12 (one of the correctly targeted mice) because this mouse died before weaning, and enough genomic DNA was not available. We sequenced the targeted region of the three non-targeted founders (2, 5, and 9) to check if they contained indels and found that they all had indels (data not shown). This suggested that the genome editing efficiency of the gRNA (Mmp9-Cr1) used for knocking in of mCitrine at the *Mmp9* locus was 100%; thus, this set of 11 mice was a good set for OT and RI analyses.

### Identification (or ruling out) of OT sites using the CRISPR-KRISPR method

**Fig. 2:**
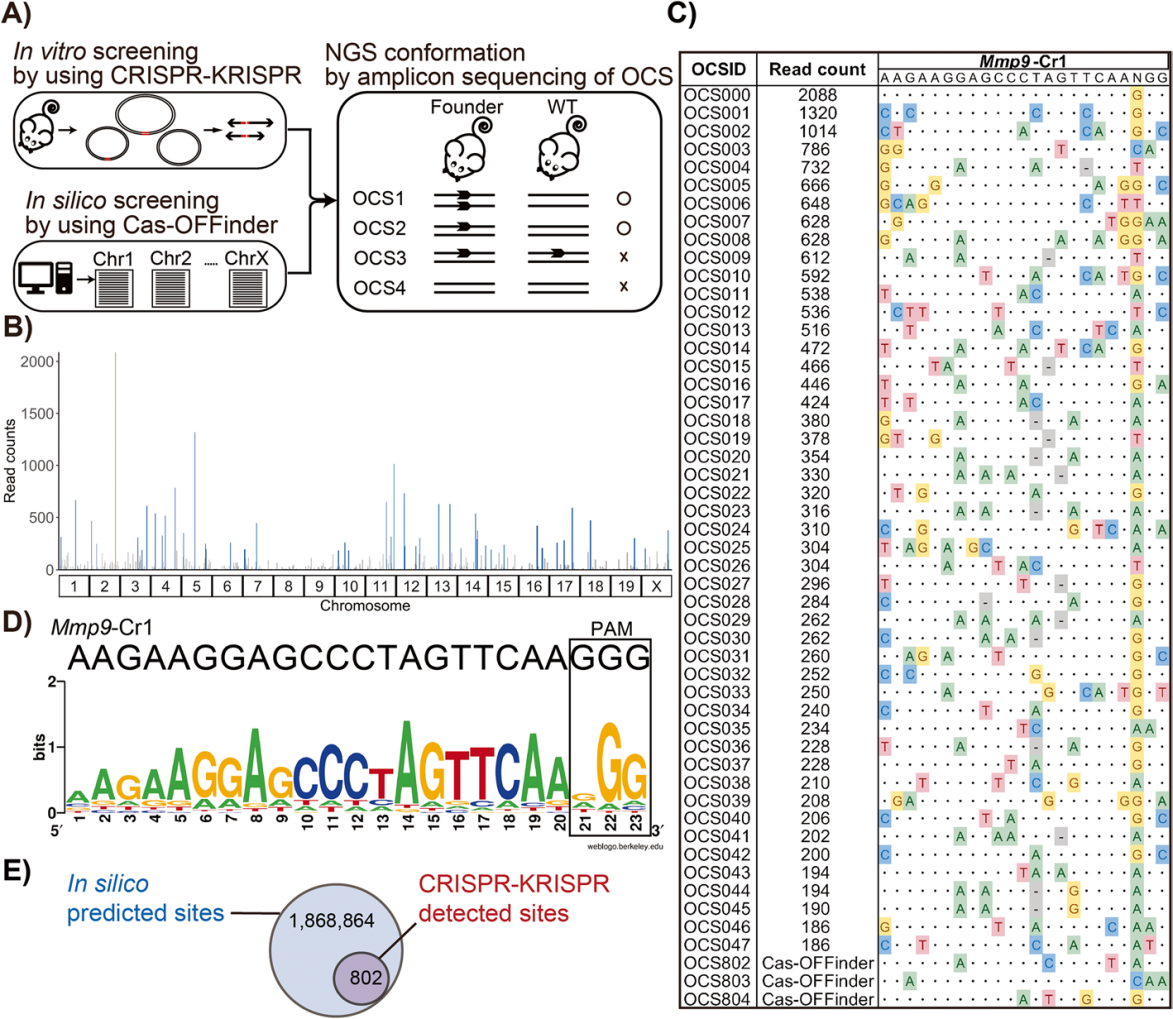
Assessment of in vivo OT indels introduced with Mmp9-Cr1 gRNA. **A** Schematic illustrating OT candidate sites (OCSs) identification and their confirmation procedure. OCSs for Mmp9-Cr1 gRNA were identified by CRISPR-KRISPR and by Cas-OFFinder. The fragments containing OCSs selected were PCR amplified from founder and wildtype (WT) mice. Amplicon sequencing of these fragments were performed using a high-throughput sequencer. **B** Manhattan plot of 802 cleavage sites detected by CRISPR-KRISPR. The length of bars represents CRISPR-KRISPR read count. The Mmp9-Cr1 on-target site is indicated in red. Top 47 OCSs are indicated in blue. **C** The top 47 OCSs detected by CRISPR-KRISPR and three predicted OCSs by Cas-OFFinder are shown. **D** Sequence logo of cleavage sites detected by CRISPR-KRISPR. **E** Venn diagram showing the number of OCSs and overlap between those OCSs predicted by Cas-OFFinder and CRISPR-KRISPR

To investigate whether OT mutations were caused in zygote genome editing using Mmp9-Cr1 gRNA (Fig. 1 and Additional file 1: Table S1), and to identify the OT candidate sites (OCSs) (Fig. 2A), we employed the CRISPR-KRISPR method and an in silico method (Cas-OFFinder). The NGS sequencing of the CRISPR-KRISPR library identified 802 potential SpCas9 nuclease cleavage sites. The highest number of read counts was found at the on-target site of Mmp9-Cr1 (2,088 reads of 36,858 CRISPR-KRISPR identified reads) (Fig. 2B, C). In addition, the consensus sequence analysis of 802 potential cleavage sites showed a 100% match with the target sequence of Mmp9-Cr1 (5′-AAGAAGGAGCCCTAGTTCAAGGG-3′), as expected. Furthermore, it had the canonical PAM sequence of SpCas9 (5′-NGG-3′) (Fig. 2D).

Simultaneously, we used Cas-OFFinder method to predict sequence homology-based potential OT sites, which showed 862,287 and 1,006,577 potential OT sites, with canonical 5′-NGG-3′ PAM and non-canonical PAM sequences, respectively (Additional file 1: Table S2). Of note, the Cas-OFFinder list included all of the 802 potential cleavage sites identified by CRISPR-KRISPR method (Fig. 2E).

These results suggest that the CRISPR-KRISPR method can reliably identify potential OT sites.

### Evaluation of OCSs by in vitro digestion

Next, we examined whether the potential OT sites will get cleaved by Cas9 using an in vitro digestion method. For this, we chose a total of 50 sites: the list contained the top 47 (of the 802) sites identified from the CRISPR-KRISPR approach (i.e., the ones that had highest read counts), and the remaining three sites were predicted by in silico analysis (these sites had three or fewer mismatches) (Fig. 2C). We designed PCR primer sets (Additional file 1: Table S3) to amplify using the wildtype genome as a template, followed by Cas9 treatment of the PCR fragments using Mmp9-Cr1 gRNA. Cas9 nuclease cleaved 7 of the 50 sites (OCS#001, OCS#011, OCS#017, OCS#022, OCS#025, OCS#028, and OCS#037) (Fig. S2). The cleavage efficiency of on-target sites was 94.7%, and those of OCSs (7 sites) ranged from 13.7 to 70.9%. All of the cleaved OCSs had canonical PAMs.

### Validation of OT mutations by targeted amplicon sequencing

**Fig. 3:**
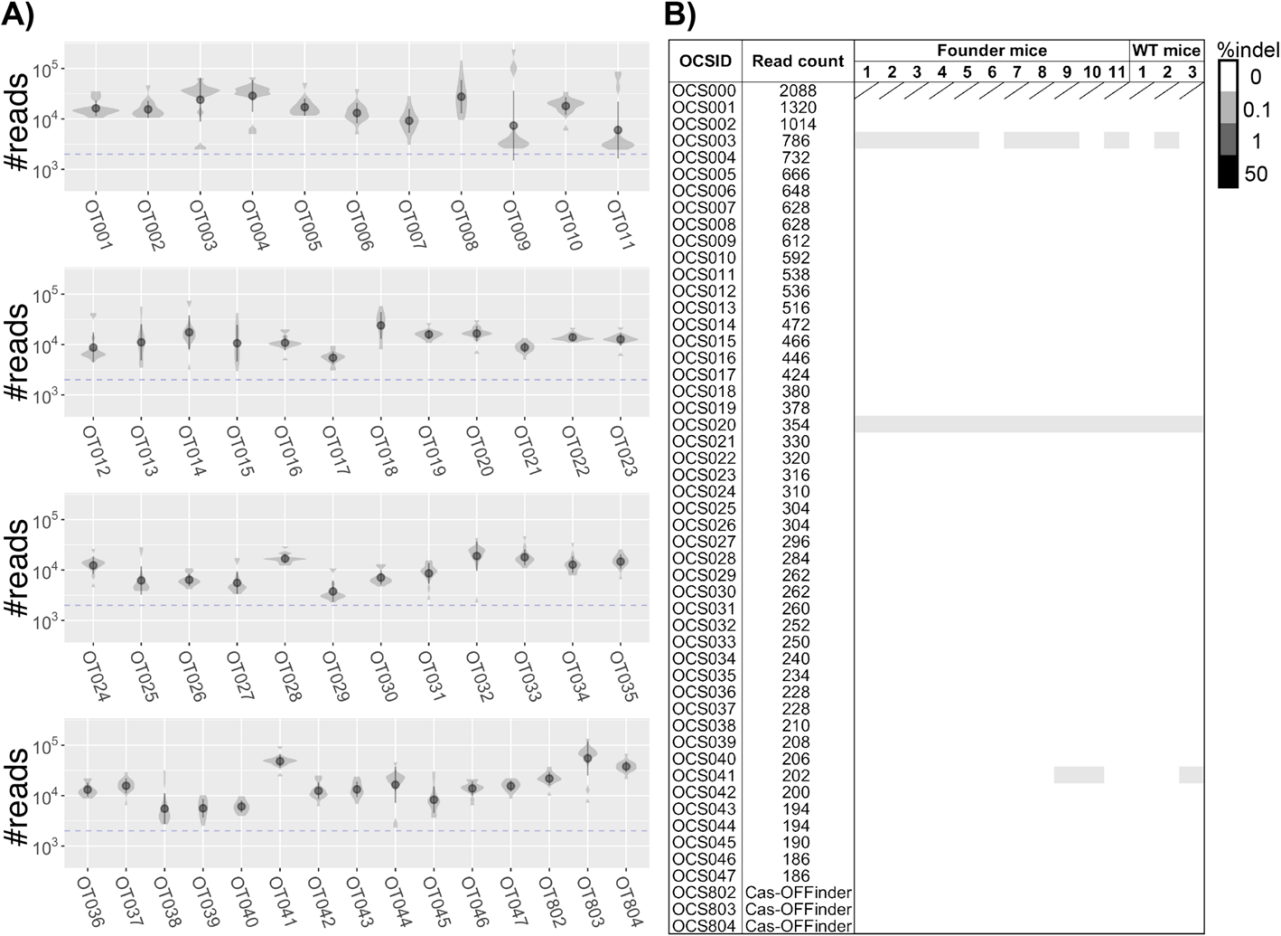
Validation of in vivo OT indels using targeted deep amplicon sequencing. **A** Targeted deep amplicon sequencing for OCSs. Violin plots show the distribution of the number of reads for each OCS (top 47 OCSs detected by CRISPR-KRISPR (OCS001~OCS0047) and three uniquely predicted sites by Cas-OFFinder (OCS802~OCS804)). Median distributions are shown by dark grey dots and lines, respectively. The dashed line indicates required minimum number of reads (2,000 reads). **B** Validation of 50 OCSs in 11 founder mice and three wildtype (WT) mice

To confirm whether the Mmp9 reporter founder mice carried OT mutations in these 50 OCSs, we performed targeted amplicon sequencing of all the 11 founder mice (Fig. 3A). The results showed that the maximum frequency of indel mutations detected was less than 0.2%, and these mutations were detected in amplicons of both F0 and wildtype mice. If the mutation is introduced by four-cell stage, the frequency of detection of the mutant allele is expected to be ~12.5%. However, no indel mutations exceeded this rate (Fig. 3B). In vivo OT mutations were also undetected in the seven sites where cleavage was confirmed by in vitro digestion. Thus, we conclude that Mmp9-Cr1 gRNA did not cause OT mutations at 50 OCSs above a detection limit of approximately 0.1%.

### Leveraging the CRISPR-KRISPR method to identify RI sites

In our previous knock-in reporter mouse model generation experiment (*Mmp9*-T2A-mCitrine) [6], we noted one of the untargeted founders contained randomly inserted donor DNA. Founder mouse 9 was negative for 5′ and 3′ junction PCRs, but the internal primers (that bind only to the donor DNA sequence) amplified an expected sized band (Additional file 1: Fig. S1B and S1C). This suggested that the T2A-mCitrine cassette may have gotten inserted at a random genomic region (see founder 9 in Additional file 1: Fig. S1C). In order to assess additional RI events among the entire panel of founders, we estimated the donor copy number in each founder mouse by qPCR analysis using internal primers (that bind to the insertion cassette T2A-mCitrine). This assay is expected to show either a value of 1 (mono-allelic or heterozygous insertion) or 2 (bi-allelic or homozygous insertion), assuming no mosaicism. If the value is more than 2, it suggests that the founder animal may contain additional insertions (elsewhere in the genome). Note that a value of 2 may also indicate one correctly targeted allele (heterozygous) and another random insertion. The results showed that founder 7 contained more than three copies (Additional file 1: Fig. S1D), suggesting that this mouse may also have RI (or multiple copies are inserted on target in a tandemly connected manner). We then set out to systematically analyze RIs among all of the 11 founder mice using the CRISPR-KRISPR method.

The strategy we employed is as follows. We designed two gRNAs—gRNA-L and gRNA-R—to use them in the CRISPR-KRISPR assay. The gRNA-L binds to the left arm whereas the gRNA-R binds an overlapping region of the right arm and the insertion cassette (Additional file 1: Fig. S3A and Table S1). CRISPR-KRISPR libraries derived from pooled genomic DNA from all 11 founder mice (including genomic DNA from a wildtype mouse as a separate sample) were treated with a respective gRNA, and the regions cleaved by Cas9 were sequenced by next-generation sequencing (NGS) to identify insertion candidate sites (ICSs) (Additional file 1: Fig. S3B). As a result, 8 and 47 cleavage sites were detected for gRNA-L and gRNA-R, respectively. These cleavage sites were confirmed by the Integrative Genomics Viewer (IGV), which revealed 26 ICSs (Table 1). Among these, ICS#1, ICS#2, and ICS#21 were thought to be derived from the on-target region (Table 1). Based on these analyses, we identified a total of 23 ICSs (Table 1). Intriguingly, 17 out of 23 ICSs were detected in protein-coding genes (mostly in introns). Of note, none of those 23 ICSs were located at the potential OCSs described above (Additional file 1: Fig. S4).

**Table 1:** Summary of ICS detected by CRISPR-KRISPR

Insertion candidate sites (ICS)	gRNA used	No. of reads	Founder mouse	Chromosome	Start	End	Genomic region
#1	right	1820	-	2	164954903	164955063	*Mmp9* on target
#2	left	1680	-	2	164954709	164955052	*Mmp9* on target
#3	right	269	6	2	43676988	43677099	*Kynu* intronic region
#4	right	158	1	2	164958347	164958445	Intergenic region
#5	right	74	7	2	27945902	27946004	*Col5a1* intronic region
#6	right	51	-	10	8621100	8621200	*Cnnm2* intronic region
#7	left	48	9	9	63264772	63264909	*Map2k5* intronic region
#8	right	43	-	6	91473078	91473209	*Chchd4* intronic region
#9	left	42	7	2	27947501	27947631	*Col5a1* intronic region
#10	right	40	11	2	164951625	164951696	*Mmp9* intronic region
#11	right	30	-	11	44994980	44995104	*Ebf1* intronic region
#12	left	25	-	12	118243649	118243801	*Sp4* intronic region
#13	right	23	-	15	86334151	86334255	*Tbc1d22a* intronic region
#14	left	23	6	2	43677155	43677290	*Kynu* intronic region
#15	right	21	11	2	164951440	164951518	*Mmp9* intronic region
#16	right	20	-	17	10769843	10769900	*Pacrg* intronic region
#17	right	20	-	17	61954898	61955001	Intergenic region
#18	right	15	-	10	107374530	107374593	*Lin7a* intronic region
#19	left	15	-	6	38606367	38606454	*Luc7l2* exonic/intronic region
#20	right	12	-	18	43026996	43027098	*Ppp2r2b* intronic region
#21	right	10	-	2	164954849	164954903	*Mmp9* on target
#22	right	8	-	1	88986042	88986123	Intergenic region
#23	right	8	7	19	57114454	57114530	*Ablim1* intronic region
#24	right	8	-	6	10123095	10123157	Intergenic region
#25	left	7	-	5	32180060	32180151	Intergenic region
#26	right	6	-	2	74223656	74223761	Intergenic region

### Attributing different ICSs, which were identified by CRISPR-KRISPR analysis of the pooled genomic samples, to specific founders of the panel

**Fig. 4:**
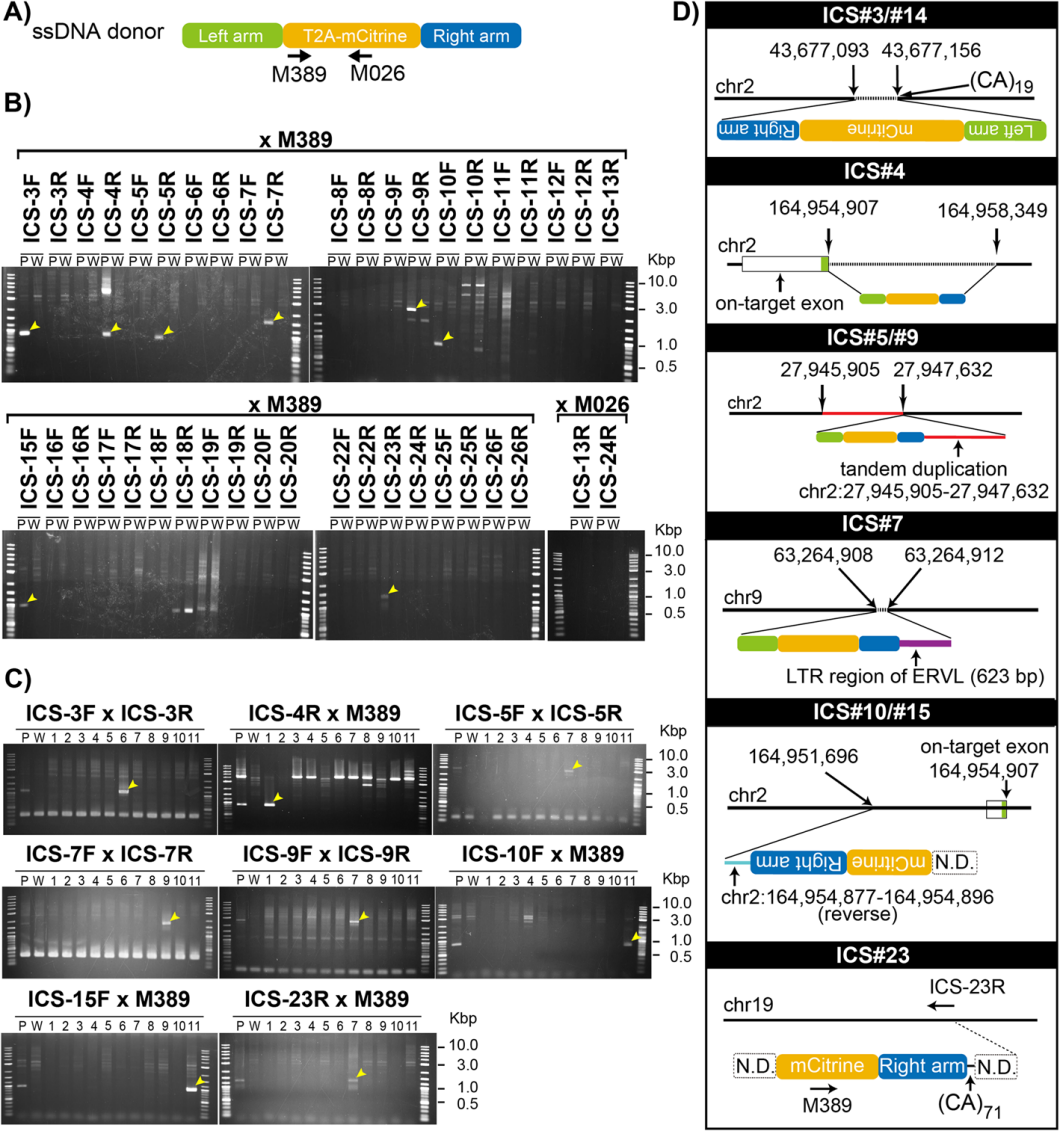
Detection and characterization of RIs and imprecise on-target insertions of the donor DNA in founder mice. **A** Location of PCR primers (M389 and M026) in the donor DNA cassette. **B** PCR screening of insertion sites for 22 ICSs using the pooled DNA derived from founder mice (P) and wild type (W) as templates. Yellow arrows indicate the fragments amplified only from founder mice. **C** PCR screening of the individual founder mice for eight ICSs detected by PCR in (**B**). Yellow arrows indicate the fragments uniquely amplified from founder mice. **D** Schematic for configuration of the inserted sequence. The junctional sequences of six loci (from nine ICSs) were analyzed by Sanger sequence. Chromosome positions were obtained from the UCSC Mouse Genome Browser, mm10 assembly. (CA)_19_ and (CA)_71_ indicate the length of 19 and 71 CA repeats, respectively. Dashed horizontal lines indicate deleted region of the genome. The solid red line indicates the tandemly duplicated region with mCitrine cassette. The purple line indicates a long terminal repeat (LTR) region of the endogenous retrovirus (ERVL). N.D., not determined

The CRISPR-KRISPR analysis was performed using the pooled DNA samples from all of the 11 mice generated in a microinjection experiment. To experimentally verify the ICSs, and to identify which ICSs corresponded to which founders, we designed PCR primers (ICS primers) for all the ICSs (note that we designed 22 primer sets for 23 ISCs) (Additional file 1: Table S4); ICS#3 and ICS#14 were considered to be derived from the same ICS based on the mapped sequence (Additional file 1: Fig. S5). PCR was performed using pooled gDNA containing equimolar amounts of gDNA from 11 founder mice and a wildtype mouse as templates to verify the presence of the insertion cassette (Fig. 4A). Amplification of PCR fragments was detected at 8 out of 22 sites (Fig. 4B). Next, to identify founder individuals with RI sequences, PCR was performed for each mouse for these eight independent sites. The results showed that PCR amplification detected the eight sites in 5 of the 11 F0 mice (founders 1, 6, 7, 9, and 11) (Fig. 4C). None of the insertions were the same among different founder mice.

The insertion positions of all the eight sites were identified and confirmed by Sanger sequencing. ICS#3/#14 were identical, and the mCitrine cassette was inserted into the CA repeat sequence of the *Kynu* intronic region, accompanied by a 62 bp gap. In ICS#4, the mCitrine cassette was inserted at the on-target site with an approximately 4 kb deletion. ICS#5/#9 was also an identical ICS, with the insertion of an mCitrine cassette accompanied by a duplication of an approximately 1.7 kb region adjacent to the insertion site. ICS#7 had an mCitrine cassette insertion, along with 623 bp of a long terminal repeat (LTR) sequence of murine endogenous retrovirus (ERVL). The results of PCR analysis suggested that the mCitrine cassette was inserted in the intronic region of the *Ablim1* gene in the reverse direction in ICS#23. However, the flanking sequence could not be determined due to the presence of more than 71 repeated CA repeats immediately downstream of the right arm (Fig. 4D). The CRISPR-KRISPR analysis identified ICS#10/#15, which is also an identical ICS located close to the on-target site of founder mouse 11, and the sequence was confirmed up to the middle of the T2A-mCitrine cassette, but the rest of the sequence could not be determined. Further analysis of this founder mouse 11 revealed a complex mix of mosaic alleles. In brief, this study could identify four different alleles, each with imperfections at either or both ends of the insertion sites. The results of this mouse necessitated a separate discussion, which is included as supplementary text (Additional file 1: Supplemental text). If this mouse were chosen for breeding to establish germ line transmission, correctly targeted alleles would never have been found in the F1 offspring because such an allele did not exist in this founder mouse. The portions of alleles containing correct ends at the junctions perhaps contributed to showing correctly targeted PCR amplicons when standard junctional-PCR genotyping assays were performed.

### Characterization of ICSs identified by the CRISPR-KRISPR method

Of the 23 ICSs identified, nine sites (at six different loci as described above) were found to contain mCitrine cassettes. Of the six loci, two were derived from inaccurate insertions into the target or the close vicinity of the on-target region (ICS#4 and ICS#10/#15), and the remaining four (ICS#3/#14, ICS#5/#9, ICS#7, and ICS#23) were derived from randomly inserted cassettes in places unrelated to the on-target region. It is noteworthy that all these randomly inserted loci were found to be in the intronic region of the gene. As for the 14 ICSs for which actual insertions could not be confirmed, a possibility of RI of cassettes cannot be completely ruled out, and it is interesting to note that nine of them were mapped within introns of known genes. We then examined chromatin accessibility at 23 ICSs using genome-wide DNase I hypersensitive sites (DHSs) data in early mouse embryonic stages [30] to see if genes in these regions were expressed (in those embryonic stages) and had open chromatin structures. The results showed no ICSs overlapping with the DHSs in all embryonic stages analyzed, i.e., one-cell, two-cell, four-cell, eight-cell, and Morula stages (Additional file 1: Fig. S6). We also assessed the expression profiles of 13 genes in association with 23 ICSs based on RNA-seq data of early mouse embryos (one-cell, two-cell, and four-cell stages) registered in DBTMEE (http://dbtmee.hgc.jp/index.php). However, we did not find any association between the gene expression patterns at the one-cell, two-cell, and four-cell stages and ICSs (Additional file 1: Fig. S7).

Next, we performed Dotplot analysis of the four RI loci (ICS#3/#14, ICS#5/#9, ICS#7, and ICS#23) to examine if sequence similarity between the surrounding sequences and the insertion cassettes existed. The analysis was performed using the genomic similarity search tool YASS (https://bioinfo.lifl.fr/yass/yass.php). The results showed that sequences in the vicinity of two ICSs (ICS#3/#14 and ICS#5/#9) had partial homology to the mCitrine cassette sequence (Additional file 1: Figs. S8-S9).

Since three of the four insertions (ICS#3/#14, ICS#7, and ICS#23) were detected within or accompanied by repeat sequences (microsatellite, LTR of ERVL, and microsatellite, respectively, as described above), we hypothesized that the insertions tend to occur at higher frequencies near the repeat sequences. To verify this, we used Dfam (https://dfam.org/home) to investigate the presence of repetitive sequences within the neighboring sequences in all four loci. The results showed that all loci contained one or more repetitive sequences in the flanking region (around 500 bp) to varying degrees: ICS#3/#14 was inserted near LTR, short interspersed nuclear element (SINE) and simple repeat sequences (corresponding to 69.0% sequence length), ICS#5/#9 was inserted near SINE and Simple repeat sequences (corresponding to 6.5% of the sequence length), ICS#7 was inserted near SINE and long interspersed nuclear element (LINE) sequences (corresponding to 24.6% of the sequence length), and ICS#23 was inserted near SINE sequences (corresponding to 13.1% of the sequence length) (Additional file 1: Figs. S10-S13). The repeat elements such as LINEs (19.2%), SINEs (8.2%), LTRs (9.9%), and Simple repeats (2.3%) occupy about 41.2% of the mouse genome [31]. These results suggest that although we noted some RIs were in the repeat sequences, or accompanied by repeat sequences, in general, it is hard to conclude whether RIs always occur near repeat sequences. Even if RIs are accompanied by repeat sequences, they are not always in the regions where repeat sequences are abundant.

## Discussion

### Modification of the CIRCLE-seq protocol

In this study, we made modifications to the original CIRCLE-seq protocol to identify OT and RI sites among the CRISPR knock-in mice and we named the method CRISPR-KRISPR. One of the drawbacks of CIRCLE-seq is that it requires as much as 25 μg of DNA. Although we initially used the original protocol, the yield of library DNA was too low to be used for analysis. Changing the genome fragmentation step to an enzymatic cleavage method using KAPA HyperPlus increased the yield of library DNA by about 150-fold. This allowed us to perform CIRCLE-seq with an amount of ~3 μg of genomic DNA; this amount can be easily obtained from tail piece or earpiece DNA isolations. The CRISPR-KRISPR method also has the advantage that it does not require equipment for DNA fragmentation. One of the aspects of enzymatic fragmentation approaches is that they may induce the formation of library molecules containing regions of nearby DNA from opposite strands (<5%), which may need to be kept in mind during analysis [32]. Recently, Lazzarotto et al. reported a CHANGE-seq method that can reduce the amount of DNA required to 5 μg by efficiently generating a circularized library using the Tn5 transposase. In theory, CHANGE-seq can be potentially used for identification of RI sites [33].

### The OT mutations in the knock-in mice were below the detection limit and were insignificant, as reported previously in the literature

The OT mutation frequency observed by the targeted deep amplicon sequence was close to the NGS error rate (~0.1%). Since it is difficult to detect low frequency mutations of less than 0.1% in amplicon sequencing [34, 35], and since these low frequency mutations have been observed in amplicons of both founder and WT mice, these mutation candidates are considered to be background noise, such as PCR errors or sequencing errors. Although the present analysis is based only on an experiment using one gRNA (Mmp9-Cr1), it suggests that this gRNA is not likely to introduce OT mutations in mouse embryos. This is consistent with previous reports that OT mutations are seldom introduced in mouse-embryo injection experiments [15, 16].

### CRISPR-KRISPR can be used for identifying (or ruling out) RI sites

The CIRCLE-seq method was originally developed for OT analysis of gRNA cleavage. In this study, we show that a modified version of CIRCLE-seq (CRISPR-KRISPR) can also be used for RI sites of fragments by designing gRNAs in the sequences binding to the donor DNA. RI analysis by CRISPR-KRISPR can also be used for analyzing founders in the following types of genome engineering experiments: insertion of tag sequences such as loxP into the genome, insertion of AAV vectors in gene delivery experiments, and insertion of donor DNA fragment by newer CRISPR-based methods such as prime editing. The CRISPR-KRISPR method helps identify, or rule out, both OT and RI as a unified method by using several gRNAs in the experiment: the knock-in gRNA for OT detection and gRNA(s) binding to the donor DNA for RI detection, respectively. Thus, the method can be adapted as a standard practice to evaluate founder generation animals prior to their establishing the germ line-transmitted breeder lines. The CRISPR-KRISPR method can also be useful for characterizing knock-in loci in other model organisms, including cell lines [36].

When designing gRNAs for ICS detection, the distance and position from the arm end of the insertion cassette to the gRNA must be considered, which can be dependent on the NGS read length. We used an lssDNA knock-in project as a test case to evaluate the CRISPR-KRISPR method because the donor cassette contains shorter homology arms (about 60 bases long). This knock-in project was amenable for identifying RI events using a short-read sequencer like MiSeq that reads up to 150 bases. Also, the presence of repeat sequences such as LINEs in the vicinity of the RI position can also make detection difficult using the short-read sequencing method. In case of situations like these (donors that contain longer homology arms or sequences with repeat regions), the MiSeq-based short-read sequencer approach may not be useful. However, there has been significant technical advance during the past couple years in long-range sequencing (LRS), which should be useful in such situations [37]. It is noteworthy that the method to identify RI sites of transgenes using Nanopore Technologies (called CRISPR-LRS) was developed recently [38].

We would like to note that CRISPR-KRISPR method can potentially identify partial insertions. It is possible that partial insertions of truncated fragments can occur in knock-in experiments. Note that we used two guides, one each in the homology arm regions, and as expected, all the RIs contained the guide binding sites. The CRISPR-KRISPR method can possibly be used for identifying partial insertions of different regions of the insertion cassette if gRNAs binding to different regions throughout the cassette are included in the assay.

### Characteristics of RI sites in the genome-edited mice

The CRISPR-KRISPR approach identified a total of 802 OT cleavage sites and 23 ICSs (Additional file 1: Fig. S4), but none of the 23 ICSs were in any of the 802 OT sites. This suggests that the donor DNA fragments were not inserted into the potential OT sites because of the Cas9 cleavage. In cell culture experiments using reporter cassettes, it is shown that RIs can occur in knock-in experiments using both ssDNA and dsDNA donors independent of target homology. Of the two formats of donors, dsDNAs are thought to have higher RI rates compared to ssDNAs [39], although we have not tested the RI rates of dsDNA donors using the CRISPR-KRISPR method. Considering that 7 out of 12 mice contained correctly targeted alleles, i.e., a twofold higher rate of correct insertions (6 out of 11 founders analyzed) compared to RI (3 out of 11), the long ssDNA insertion approach is still a highly efficient and reliable method for generating knock-in models. It should be noted that it is difficult to conclude typical ranges of RI rates of donor DNAs by testing only one locus. A systematic approach wherein several loci (about a dozen or more) tested using different formats of donors such as linear ssDNAs, circular ssDNA, linear dsDNA, or circular dsDNA need to be analyzed in order to find out the typical ranges of RI rates of different donor DNA formats.

Our results show that donor DNA insertions occurred at regions where no homology with the donor was found, although some inserted loci had partial homology to the mCitrine cassette. We also found that DNA donors tend to insert into the intronic regions and/or repetitive sequence regions. Regarding the RI of dsDNA into the genome, there are several reports on the insertion tendency. For example, among the transgenic animal models generated by conventional methods, transgene sequences typically insert into host gene regions [40], near minisatellite sequences [41], or at repeat elements such as retrotransposons [42]. It is also reported that insertions can occur in addition to DNA rearrangement [42]. In the iGUIDE method using dsDNA as a donor, it is reported that donor DNA is preferentially inserted at spontaneous DSB sites, or close to genes [43]. It is interesting to note the potential similarity of characteristics of insertion sites between dsDNA and ssDNA donor formats.

Although RIs would be a major problem in the case of somatic cell gene therapy, in general, this may not be a significant problem in knock-in animal generation because extra insertion alleles can be easily eliminated by breeding, except in the cases where the RI is very close to the on-target correction insertion. In this study, however, we identified that two of four RIs (ICS#3/#14 and ICS#5/#9) were on chromosome 2, which is the same as the on-target site. The founder mice containing these RIs (founder mice 6 and 7) also contained correct insertion at the on-target site. We have not determined whether RI and correct insertion were on the same parental chromosome in *cis*. The genetic distances between on-target (*Mmp9*) and ICS#3/#14 (*Kynu*) or ICS#5/#9 (*Col5a1*) were far enough (59.6 cM between *Mmp9* and *Kynu*, 65.9 cM between *Mmp9* and *Col5a1*) to get the respective on-target KI and RI mutations segregated by breeding. If such *cis* insertions are close to each other, breeding to segregate them may not be possible. Collectively, our observations suggest that it is important to identify founder mice that contain RIs in order to rule out lesions that are unidentifiable using standard genotyping PCRs with primers that bind to the insertional junctions [44]. Therefore, CRISPR-KRISPR can help confirm the correct on-target insertions, on-target imprecise insertions, and also rule out/identify other RIs. Once RIs are identified among the correctly targeted founders, genotyping PCRs to identify those RIs can be developed, and RI specific genotyping is required not only on the founder animals but also on the next-generation offspring to exclude the ones that contain RIs for further breeding. Since mosaicism in founders is one of the common problems among the CRISPR genome edited mice [45, 46], it would be necessary to confirm correct insertions in F1 mice and to ensure that the integrated sequence is a single copy in the genome by qPCR or ddPCR.

The scientific community is well aware that genotyping to identify correctly targeted animals among the CRISPR genome edited animals is not straightforward because of mosaicism and because the DNA repair outcomes at the CRISPR cleaved genomic sites are unpredictable. Several types of genomic rearrangements, including insertion of fragments of DNA from other genomic regions or fragments of retrotranscribed sequences can occur [47]. Even though standard junctional PCRs, used for genotyping founder animals, can indicate that an animal is correctly targeted, such results can be deceiving sometimes. Additional assays (like the one described in this study) can uncover the hidden complex genomic rearrangements. An example is founder mouse 11, which contained a mixture of four different incorrectly targeted alleles, yet the two junctional PCRs showed correct targeting because the two PCRs identified the correct amplicons, perhaps from the templates derived from different alleles. While this was the case with just one of six founders in this example, it would be prudent to ensure that the founder animal(s) chosen for further breeding indeed are accurately targeted and are devoid of OTs and RIs by analyzing them with an assay like CRISPR-KRISPR.

## Conclusions

The CRISPR-KRISPR method (a modified version of the CIRCLE-seq) requires only ~3 μg gDNA to generate a circularized DNA library, successfully identifying RI sites in the CRISPR knock-in mice. RI regions identified indicated that the DNA donor templates have a tendency to insert in the intronic regions, accompanied by genomic deletions or duplications, and/or with repeat elements. The CRISPR-KRISPR method can be adapted as a standard protocol to evaluate founder generation animals to choose the ones that do not have OTs or RIs for further breeding.

## Methods

### Genomic DNA

C57BL/6J mice and 11 founder mice obtained by *Easi-*CRISPR using a repair template containing the T2A-mCitrine cassette were used for CRISPR-KRISPR experiments [6]. Genomic DNAs were extracted from these mouse tails using the DNeasy Blood & Tissue Kit (Qiagen).

### CRISPR guide RNAs design

For screening in vivo OCS, we used the guide RNA (gRNA) for *Mmp9* (Mmp9-Cr1) (Additional file 1: Fig. S1A and Table S1) [6]. For detection of ICS in founder mice, we designed two gRNAs (named as gRNA-L and gRNA-R) by using CHOPCHOP [19] and CRISPR-direct [48] (Additional file 1: Table S1). Each gRNA was located in the left arm and right arm of the *Mmp9*-T2A-mCitrine cassette, respectively (Additional file 1: Fig. S3A).

### CIRCLE-seq library preparation

We prepared the CIRCLE-seq library according to the previously described protocol [24], with slight modification. Briefly, purified genomic DNA was sheared with the KAPA Hyper plus kit (Kapa Biosystems) to an average length of 300 bp. The fragmented DNA was end-repaired, A-tailed, and ligated to an uracil-containing stem-loop adaptor oSQT1288 5′-P-CGGTGGACCGATGATCUATCGGTCCACCG*T-3′, where “*” indicates phosphorothioate linkage. Adapter-ligated DNA molecules were then selected by eliminating molecules that did not have adaptors ligated to both ends using a mixture of Lambda Exonuclease (NEB) and *E. coli* Exonuclease I (NEB). Adapter-ligated DNA was then treated with USER enzyme (NEB) and T4 polynucleotide kinase (NEB) to expose and add 5′ phosphorylation of cohesive ends. DNA was circularized at 5 ng/μl concentration using T4 DNA ligase and treated with Plasmid-Safe ATP-dependent DNase (Epicentre) to degrade unligated linear DNA molecules. In vitro cleavage reactions were performed in a 100 μl volume with 1 x NEB 3 buffer (NEB), 90 nM SpCas9 protein (Integrated DNA Technologies), 90 nM gRNA, and 250 ng of circularized and Plasmid Safe-treated DNA. Digested products were A-tailed, ligated with a hairpin adaptor (NEBNext Multiplex Oligos for Illumina (NEB)), treated with USER enzyme (NEB), and amplified by PCR using Kapa HiFi polymerase (Kapa Biosystems). Completed libraries were quantified by qPCR using KAPA Library Quantification Kits (NIPPON Genetics Co, Ltd). The average length of libraries was calculated as 450 bp (average length of seared genome 300 bp + hairpin adaptor 30 bp + sequence adaptor 120 bp). Sequencing was performed using a MiSeq V2 reagent kit (150 bp paired end reads) on an Illumina MiSeq instrument. For identification of OCS, the library treated with Mmp9-Cr1 was sequenced 2 million reads. For identification of ICS, the libraries treated with gRNA-L and gRNA-R were sequenced 10 million and 13 million reads, respectively.

### CIRCLE-seq data analysis

To identify OCSs, the CIRCLE-seq data were processed using v.1.1 of the CIRCLE-seq open-source analysis software with the following parameters: “read_threshold: 4; window_size: 3; mapq_threshold: 50; start_threshold: 1; gap_threshold: 3; mismatch_threshold: 6; merged_analysis: True.” In this analysis, targeted sequences containing insertions (DNA bulge), or deletions (RNA bulge) compared to the gRNA strand, and non-canonical PAM for SpCas9 (such as NGA and NAG) were considered [49, 50]. The CIRCLE-seq data processing procedure for ICSs identification is illustrated in Additional file 1: Fig. S14: (1) CIRCLE-seq reads were processed by circleseq-tools with the reference-genome-independent module to identify Cas9 cleavage sites. (2) The identified reads were mapped to a T2A-mCitrine cassette sequence by BLAST search with *e*-value threshold of 1e-10 in the founder mice library. (3) After filtering, the remaining reads were mapped to mouse genome (mm10) by BLAST search with e-value threshold of 1e−10. (4) Mapped regions with less than five reads were eliminated. (5) The remaining mapped regions were manually checked by the Integrative Genomics Viewer (IGV), and the regions that passed all the filters were regarded as ICSs.

### In silico screening of potential OT sites

The Cas-OFFinder tool [18] was used to find all OCSs based on sequence homology to the Mmp9-Cr1, gRNA-L, and gRNA-R with the following parameters: allowing up to six mismatches, DNA bulge size less than 2, RNA bulge size less than 2, and non-canonical PAMs. Then, we filtered the predicted sites either equal to six mismatches in the spacer or up to two mismatches in the canonical NGG PAM.

### Targeted amplicon sequencing for OCS analysis

The primer pairs for OCS amplicon sequencing were designed using Primer3 web tools (Additional file 1: Table S3). Amplicons were amplified with KOD Fx DNA polymerase (Toyobo) or PrimeSTAR GXL DNA polymerase (TaKaRa) using 25 ng genomic DNA as a template. Amplicons from each mouse are mixed in equal amount, purified using AMPure XP beads (Beckman Coulter), and quantified by Qubit dsDNA High-Sensitivity kit (Thermo Fisher Scientific). The amplicon mixtures were end-repaired and A-tailed using the KAPA LTP Library Preparation Kit (Kapa Biosystems). Adapters for sequencing were ligated to A-tailed samples and purified by AMPure XP beads. A 13 pM sequence library solution was loaded onto the Illumina MiSeq flow cell. All amplicons were sequenced (more than 2,000 reads/amplicon: mean read coverage = 17,884 reads). Analysis of amplicon sequencing was performed using CRISPResso software v0.1.0 [51] with the following parameters: “-q 30 --ignore_substitutions --hide_mutations_outside_window_NHEJ --min_reads_to_use_region 100.” On-target primer pair for PCR and Sanger sequencing was previously described [6]. On-target amplicons were confirmed by Sanger sequencing.

### In vitro Cas9 digestion of OCS amplicons

OCS amplicons were prepared using the same conditions as targeted amplicon sequencing. A 20 μl solution containing 10 x NEB 3 buffer (NEB), Alt-R® S.p. Cas9 Nuclease (Integrated DNA Technologies), and gRNA mixture was incubated for 10 min at room temperature. Seventeen nanograms of PCR amplicon was added into this mixture and incubated for 30 min at 37 °C. Final concentration of SpCas9 and gRNA were adjusted to 100 nM. Confirmation of DNA cleavage was performed using an Agilent 2100 Bioanalyzer (Agilent Technologies) with High Sensitivity DNA Kit.

### ICS analyses

ICS primers were designed to amplify either entire ICS regions or junctional regions between the ICS and T2A-mCitrine cassette (Additional file 1: Table S4). Genomic DNA isolated from 11 founder mice were pooled in equal amount and diluted to 20 ng/μl concentration. Twenty microliters of standard reaction mixture for KOD FX Neo (Toyobo) was used for amplification of ICS regions with 20 ng genomic DNA as a template (pooled genomic DNA from founder mice and control genomic DNA from a wildtype mouse). PCR was performed with a denaturation step at 94^o^C for 2 min, followed by 30 cycles of 98 °C for 10 s, 60 °C for 30 s, and 68 °C for 1 min. Amplifications of PCR fragments were checked by 1% agarose-gel electrophoresis.

### Quantitative PCR analysis

To determine the copy number of the mCitrine cassette in the genomes of each founder mouse, we used primers to amplify a part of the mCitrine cassette (PP341: 5′-GGGTGCCCATCCTGGTCGA-3′, PP267: 5′-AGCTTGCCGTAGGTGGCATC-3′). ActB primer set was used as a normalization control (ActB-forward: 5′-CATGAAGTGTGACGTTGACATC-3, ActB-reverse: 5′-ATGATCTTGATCTTCATGGTGC-3′). For each sample, dilution series were made to 20 ng, 10 ng, 5 ng, 2.5 ng, 1.25 ng, 0.625 ng, and 0.3125 ng/well of DNA, then mixed with Fast SYBR® Green Master Mix (2×) (Thermo Fisher Scientific) and primers (0.5 ng in reaction solution). Each sample was analyzed using Quantstudio 3 (Thermo Fisher Scientific). Amplification conditions were 20 s at 95 °C, 1 s at 95 °C, and 20 s at 60 °C for 40 cycles. Data analysis was performed with the Quant Studio Design & Analysis Software v1.5.1. The threshold for all samples was set at 0.1, and a calibration curve was generated for all samples. After performing normalization with ActB, the copy number of each sample was calculated by comparing it to an eGFP transgenic mouse (the copy number was set to 2 because a homozygous individual was used) having the same sequence as the part of mCitrine.

## Supplementary Information

Additional file 1. This includes fourteen supplementary figures, four supplementary tables and supplementary text.

Additional file 2. Review history.

## Data Availability

High-throughput sequencing data in this study are available through DDBJ DRA accession number DRA013044 (https://ddbj.nig.ac.jp/resource/sra-submission/DRA013044) [52].
