# Supplementary material for: CRISPR-KRISPR: a method to identify on-target and random insertion of donor DNAs and their characterization in knock-in mice
Source: Genome Biol. 2022 Oct 25;23:228. doi: 10.1186/s13059-022-02779-8 (PMC9594901; doi:10.1186/s13059-022-02779-8)
Supplement: Supplementary file 1 — Additional file 1. This includes fourteen supplementary figures, four supplementary tables and supplementary text. [file 13059_2022_2779_MOESM1_ESM.pdf]

Additional file 1: Supplementary figures, tables and text

Supplementary Figures:

Figure S1; Schematic of T2A-mCitrine cassette Insertion into *Mmp9* locus and genotyping results of the founder mice.

Figure S2; *In vitro* digestion of OCS amplicons.

Figure S3; Overview of strategy for screening insertion candidate sites (ICSs).

Figure S4; Chromosome distribution of OCSs and ICSs.

Figure S5; Mapping of CRISPR-KRISPR reads for ICS#3/#14.

Figure S6; Physical map of ICSs and DNase I hypersensitive sites (DHSs).

Figure S7; Expression pattern of ICS-mapped genes in mouse early embryos.

Figure S8; Dotplot analysis of ICS#3/#14.

Figure S9; Dotplot analysis of ICS#5/#9.

Figure S10; Characterization of ICS#3/#14 flanking sequences.

Figure S11; Characterization of ICS#5/#9 flanking sequences.

Figure S12; Characterization of ICS#7 flanking sequences.

Figure S13; Characterization of ICS#23 flanking sequences.

Figure S14; Data analysis flowchart for detection of ICSs.

Supplementary Tables:

Table S1; CRISPR target sequences.

Table S2; Predicted number of off-target sites for Mmp9-Cr1 gRNA using in silico (Cas-OFFinder) and experimentally detected sites by the CRISPR-KRISPR assay.

Table S3; Sequences of the oligonucleotides used for OCS detection.

Table S4; Sequences of the oligonucleotides used for ICS detection.

Supplementary text: Analysis of the complex mixture of mosaic alleles in the founder 11

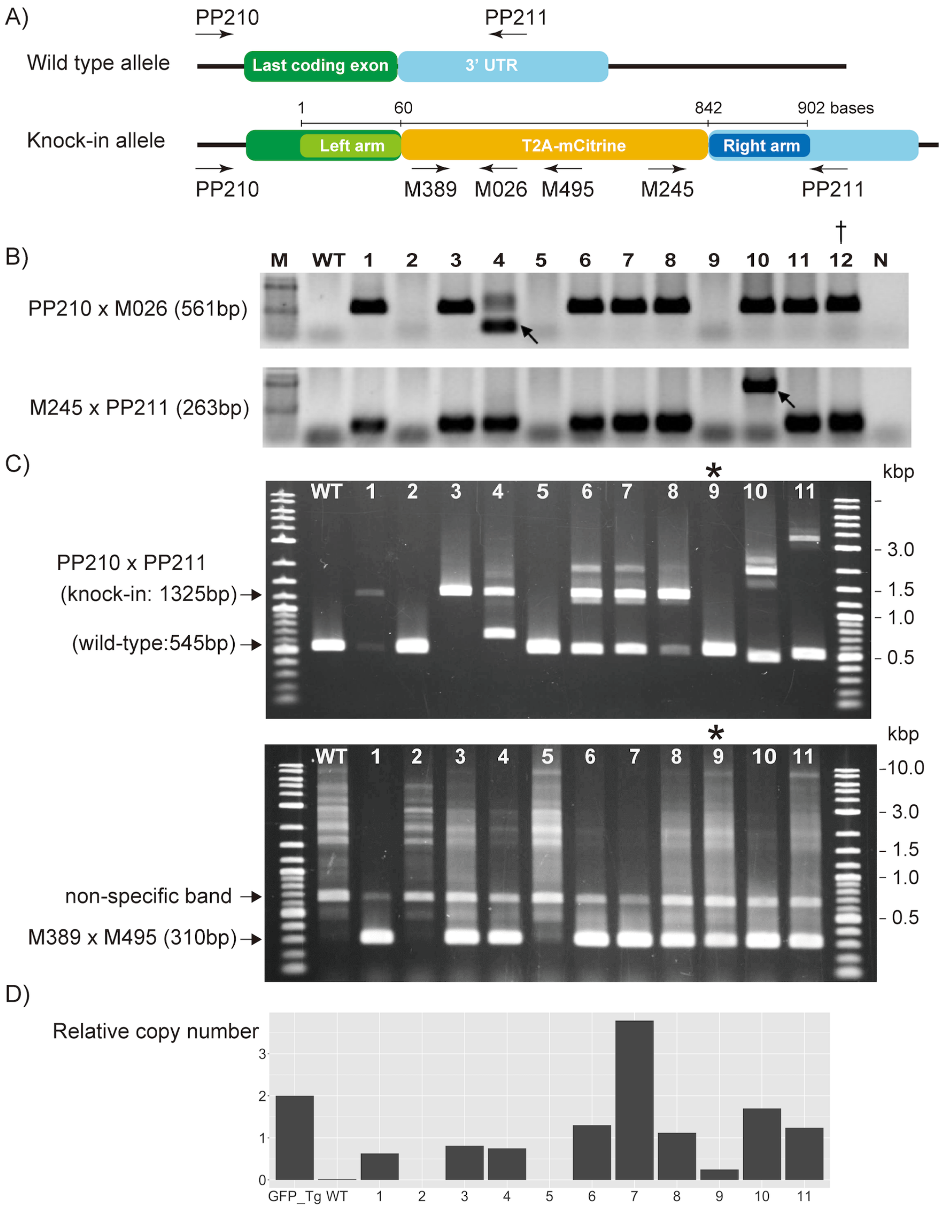

Figure S1. Schematic of T2A-mCitrine cassette Insertion into *Mmp9* locus and its genotyping results.

A) Schematic of T2A-mCitrine cassette Insertion into *Mmp9* locus using *Easi*-CRISPR. Mmp9-Cr1 cleavage site is indicated as scissors. **B)** Gel images for genotyping PCR results (data from Quadros et al 2017 [6]). † Founder 12 mouse died before weaning and was not analyzed in this study. **C)** Gel images for genotyping PCR results performed in this study. The primer sets 'PP210 x PP211' and 'M389 x M495' are for detecting 'targeted insertion' and 'target and RI', respectively. 1325 bp fragment is amplified from correct targeted insertion (knock-in) and 545 bp fragment is amplified from wild-type allele using the 'PP210 x PP211' primer set. 310 bp fragment is amplified from donor DNA cassette sequence. * Founder 9 showing no on-target insertion but containing donor cassette (suggestive of RI). **D)** Estimation of copy number for

mCitrine cassette was performed using quantitative PCR (qPCR). The copy number was calculated relative to eGFP transgenic mouse (GFP Tg). Beta actin gene was used as a normalization control.

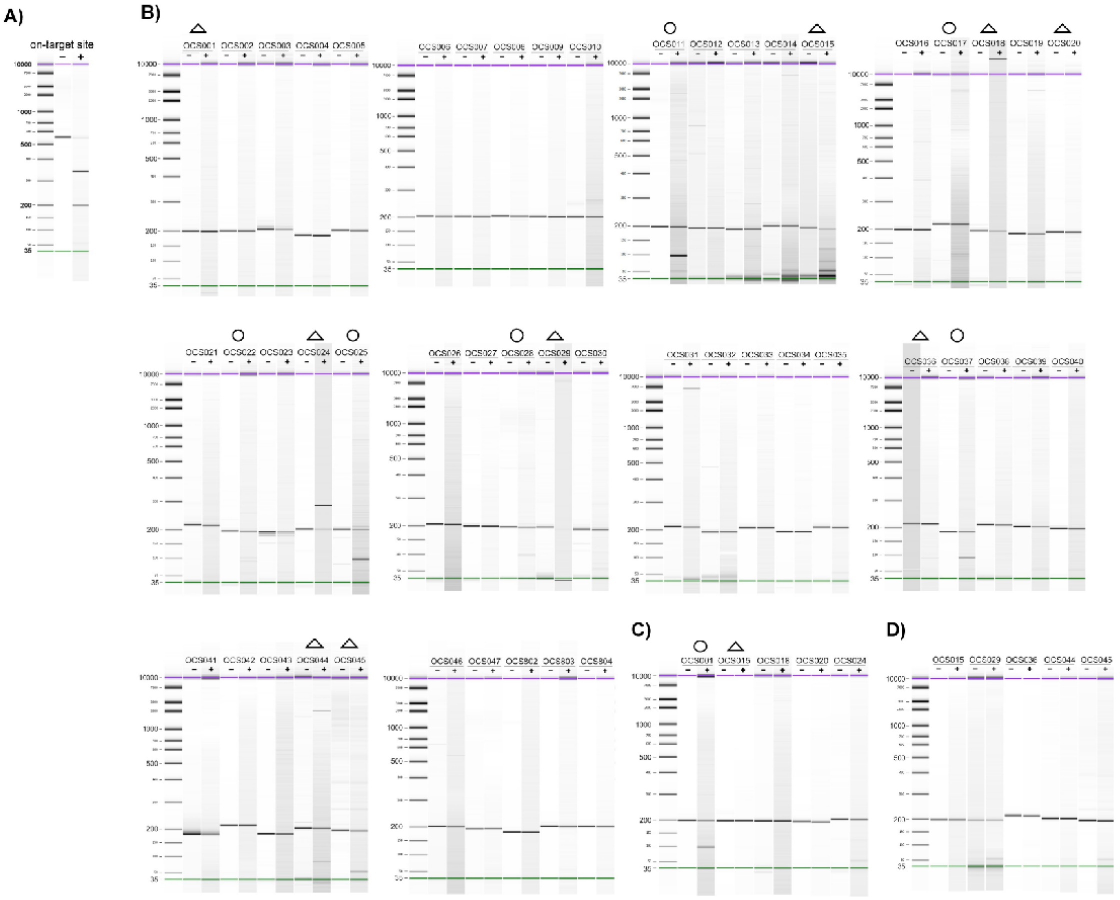

Figure S2. *In vitro* digestion of OCS amplicons.

A) *in vitro* Cas9 cleavage analyses of on-target site amplicon using Mmp9-Cr1 gRNA. (+) Cas9-treated and (-) Cas9-untreated. Molecular weight markers are shown in the left lane of each gel. B) *in vitro* Cas9 cleavage analyses of fifty OCS amplicons using Mmp9-Cr1 gRNA. Top47 sites detected by circle-seq (OCS001~OCS0047), and three sites uniquely predicted by Cas-OFFinder (OCS802~OCS804) were tested. The products showing *in vitro* cleavage are indicated as circle and products re-analyzed (in **C and D**) are shown as triangle. **C)** and **D)** *In vitro* Cas9 cleavage analyses of re-analyzed samples.

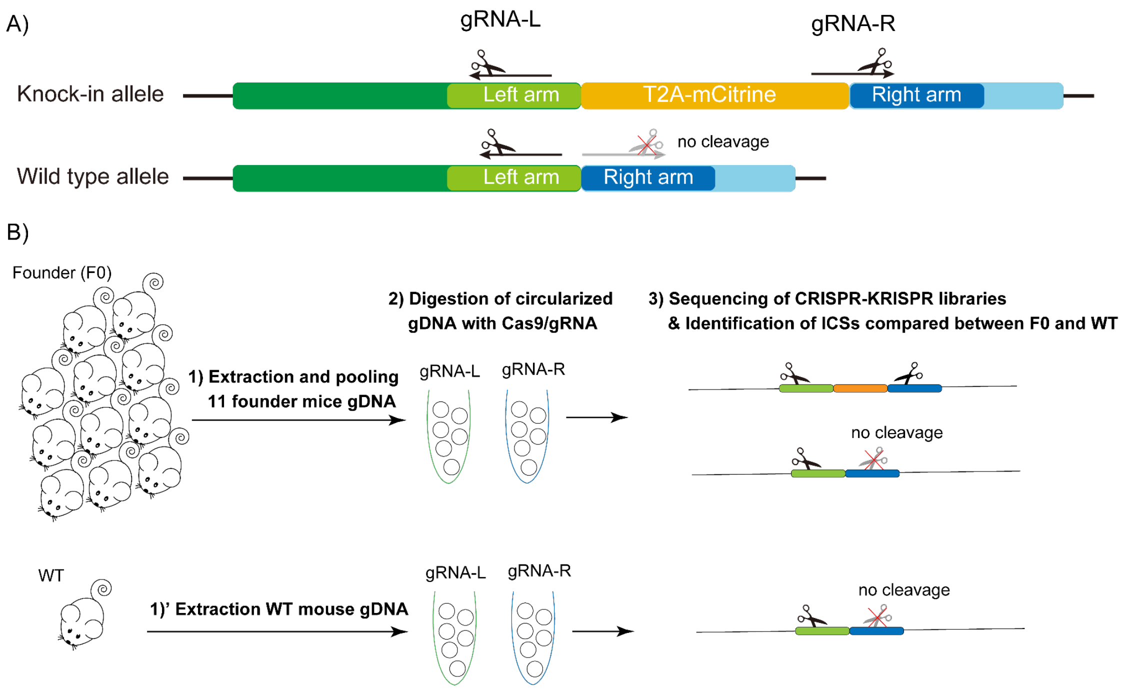

Figure S3. Overview of strategy for screening insertion candidate sites (ICSs).
A) Location of two gRNAs (gRNA-L and gRNA-R, see also Additional file 1: **Table S1**) used in the experiment. **B)** Schematic overview for detection of ICSs from eleven founder (F0) mice. 1) gDNA from eleven founder mice were extracted and equimolar pooled. gDNA from a WT mouse was extracted. All gDNAs were then fragmented and circularized. 2) The circularized DNA were treated by Cas9 together with either gRNA-L or gRNA-R. 3) CRISPR-KRISPR libraries were sequenced using high throughput sequencer to identify Cas9 cleavage sites. ICSs were detected by Circleseq-tools with reference genome-independent module.

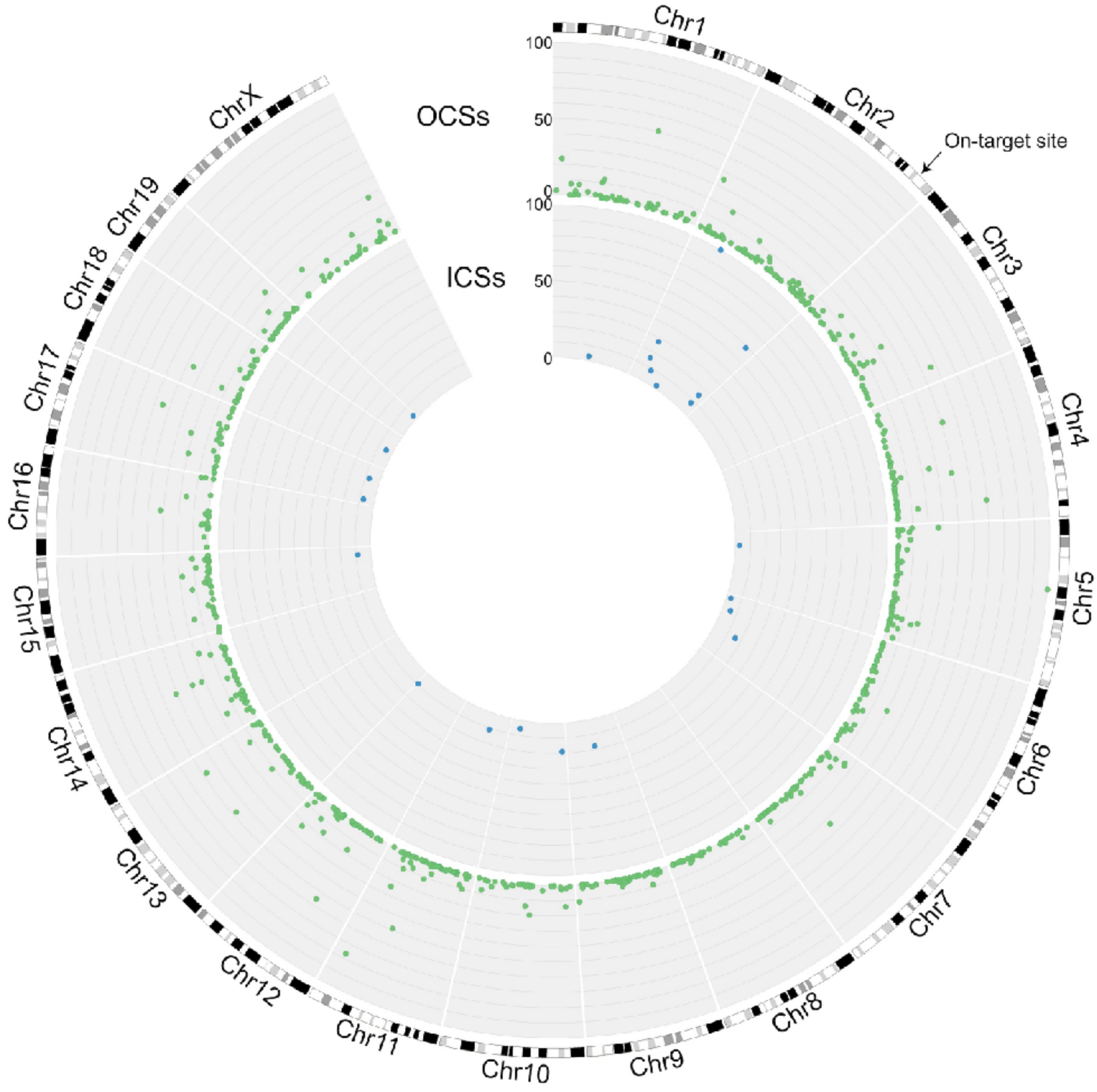

Figure S4. Chromosome distribution of OCSs and ICSs.
Scatter plots of CRISPR-KRISPR detected OCSs (excluding *Mmp9* on-target cleavage sites). The heights represent CRISPR-KRISPR read count (normalized based on the highest read count).

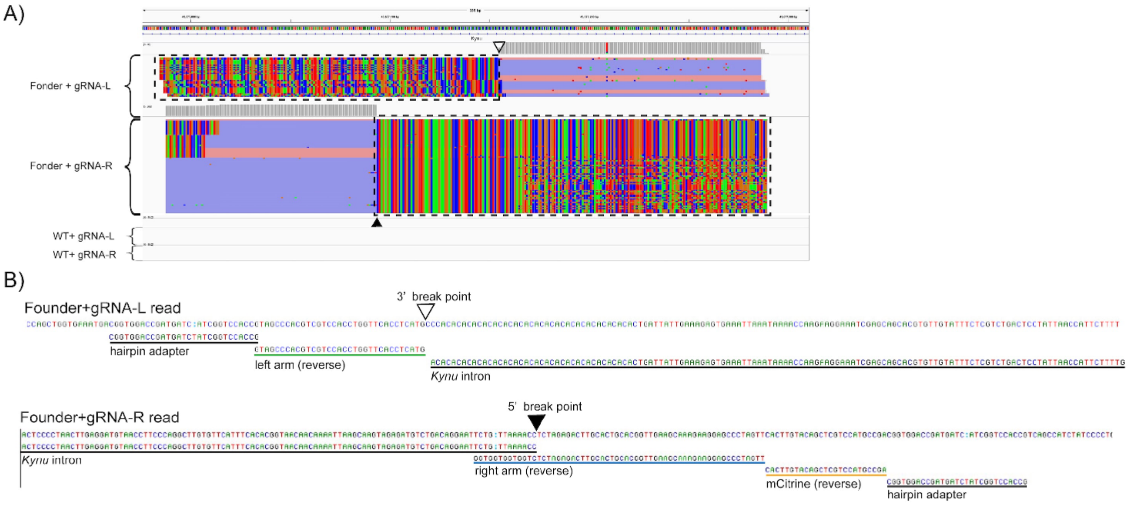

Figure S5. Mapping of CRISPR-KRISPR reads for ICS#3/#14.

A) Representative CRISPR-KRISPR reads plot in founder and wild type (WT) mice. Dashed rectangles indicate unaligned bases in partially mapped reads due to the presence of T2A-mCitrine sequence. Open and solid triangle show 3' and 5' break points of alignments against the mouse genome, respectively. **B)** Example of sequence alignment for CRISPR-KRISPR reads close vicinity of each break point (chr2:43676988-43677287, intronic region of *Kynu* gene).

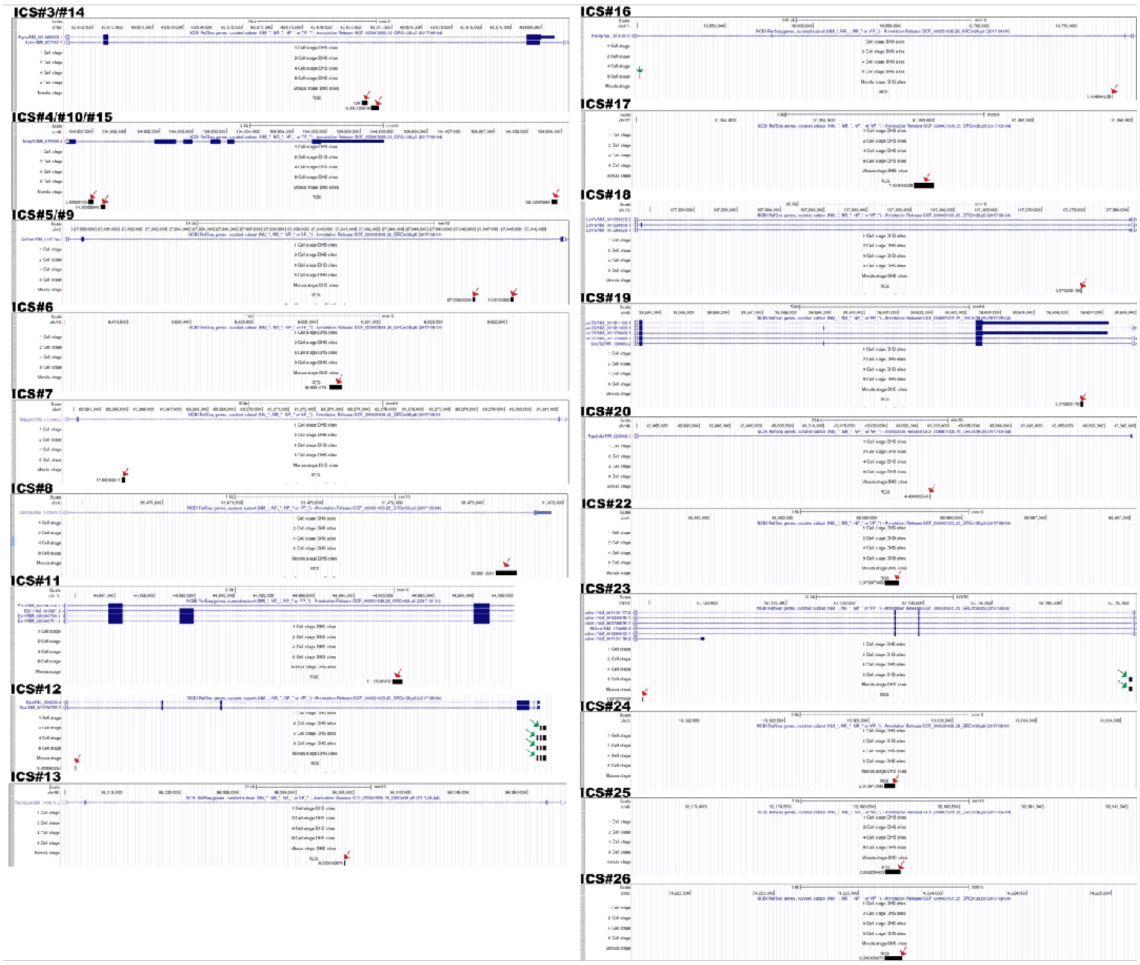

Figure S6. Physical map of ICSs and DNase I hypersensitive sites (DHSs).

23 ICSs were plotted using UCSC genome browser. DHSs dataset was retrieved from Lu et al. (2016) [30]. ICSs are indicated as red arrows. DHSs are indicated as green arrows.

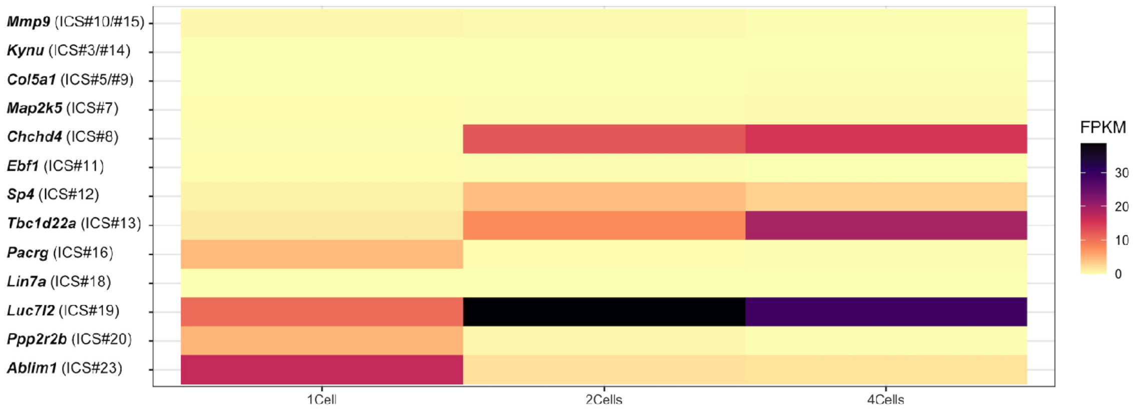

Figure S7. Expression pattern of ICS-mapped genes in mouse early embryos.
Fourteen genes where ICSs were located were plotted using 'fragments per kilobase of exon per million mapped reads (FPKM)' values. FPKM values in mouse early embryos were retrieved from DBTMEE (http://dbtmee.hgc.jp/index.php). The DBTMEE dataset (2014.4.20 update version) was used.

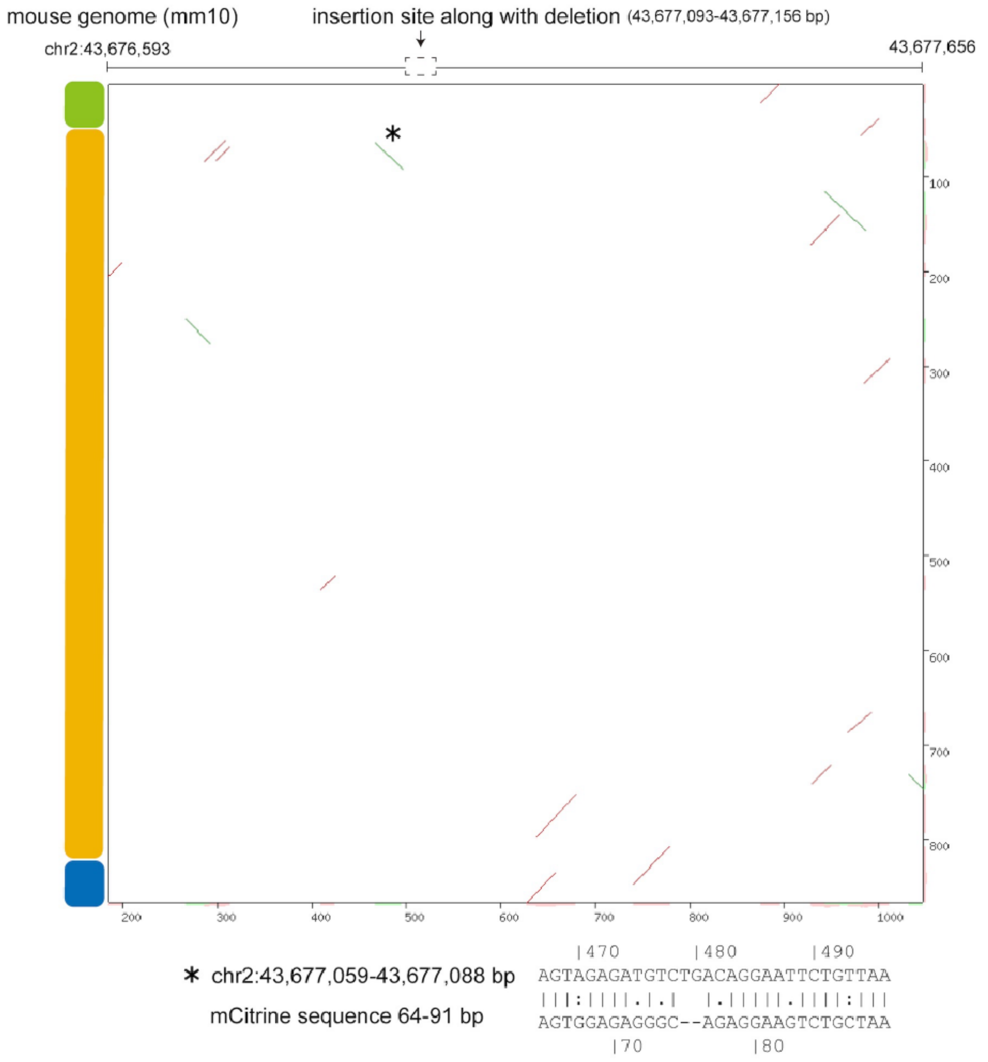

Figure S8. Dotplot analysis of ICS#3/#14.

Dot plot analysis through sequence alignment between mCitrine cassette sequence (Y axis) and reference genome (chr2:43676593-43677656) (X axis). Asterisk indicates a region close to the insertion site and showing partial homology, of which aligned sequence is shown at the bottom. Light green, orange and blue boxes indicate left arm, T2A-mCitrine and right arm, respectively.

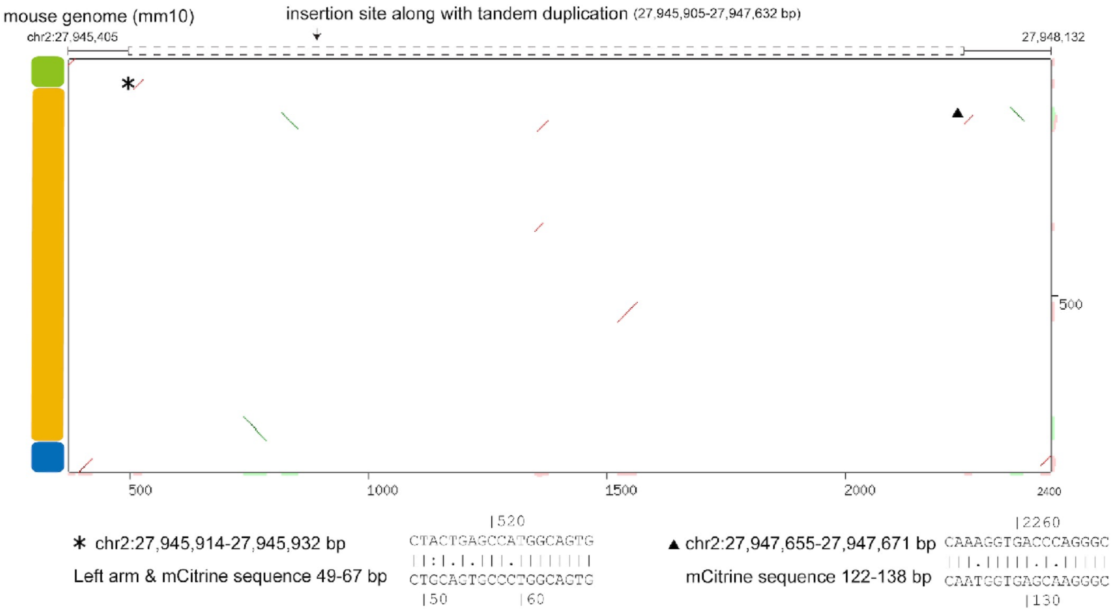

Figure S9. Dotplot analysis of ICS#5/#9.

Dot plot analysis through sequence alignment between mCitrine cassette sequence (Y axis) and reference genome (chr2:27945405-27948132) (X axis). Asterisk and triangle indicate regions close to the insertion site and showing partial homologies, of which aligned sequences are shown at the bottom. Light green, orange and blue boxes indicate left arm, T2A-mCitrine and right arm, respectively.

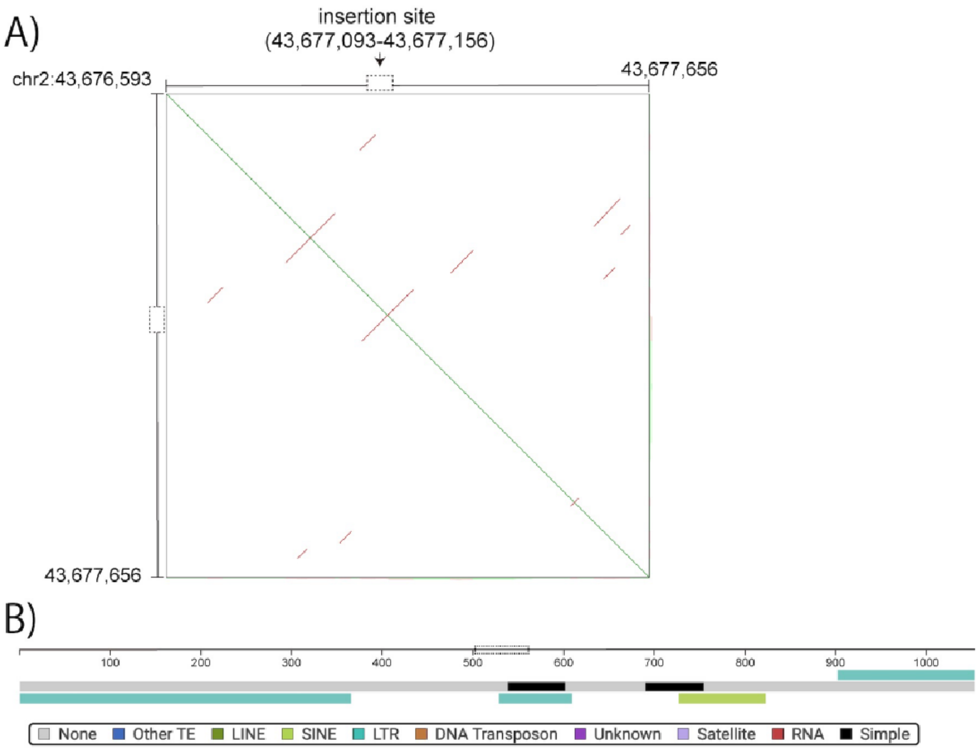

Figure S10. Characterization of ICS#3/#14 flanking sequences.

A) A dot plot indicating DNA sequence similarities, repeat sequences, inversions contained within reference sequence (chr2:43676593-43677656). **B)** Schematic view of repeat elements, predicted by Dfam, located within the ICS flanking region.

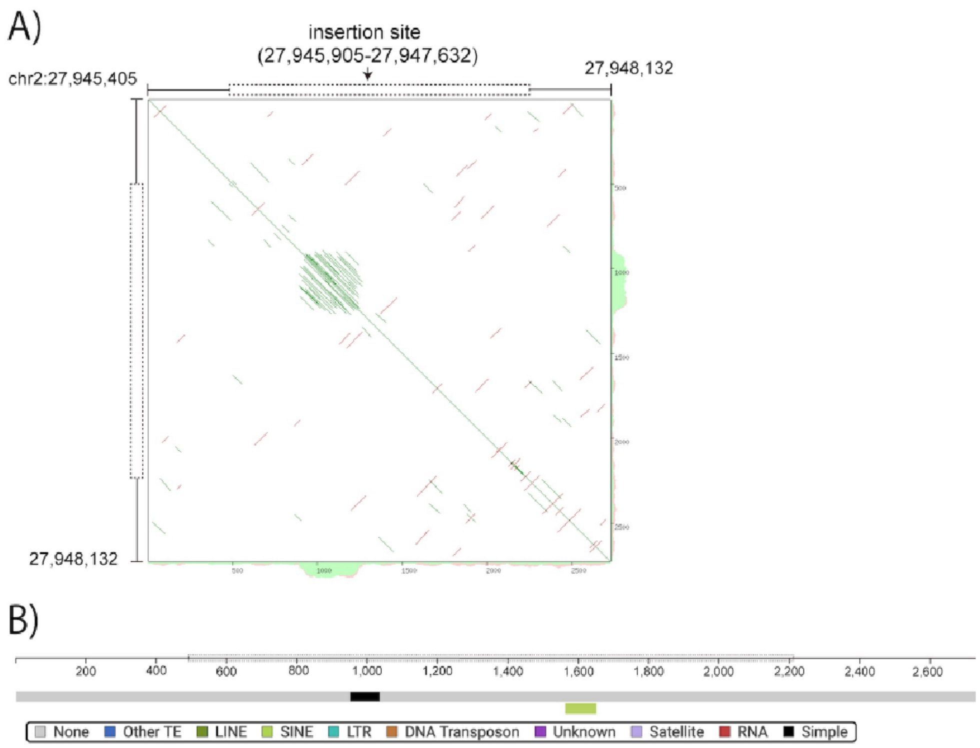

Figure S11. Characterization of ICS#5/#9 flanking sequences.

A) A dot plot indicating DNA sequence similarities, repeat sequences, inversions contained within reference sequence (chr2:27945405-27948132). **B)** Schematic view of repeat elements, predicted by Dfam, located within the ICS flanking region.

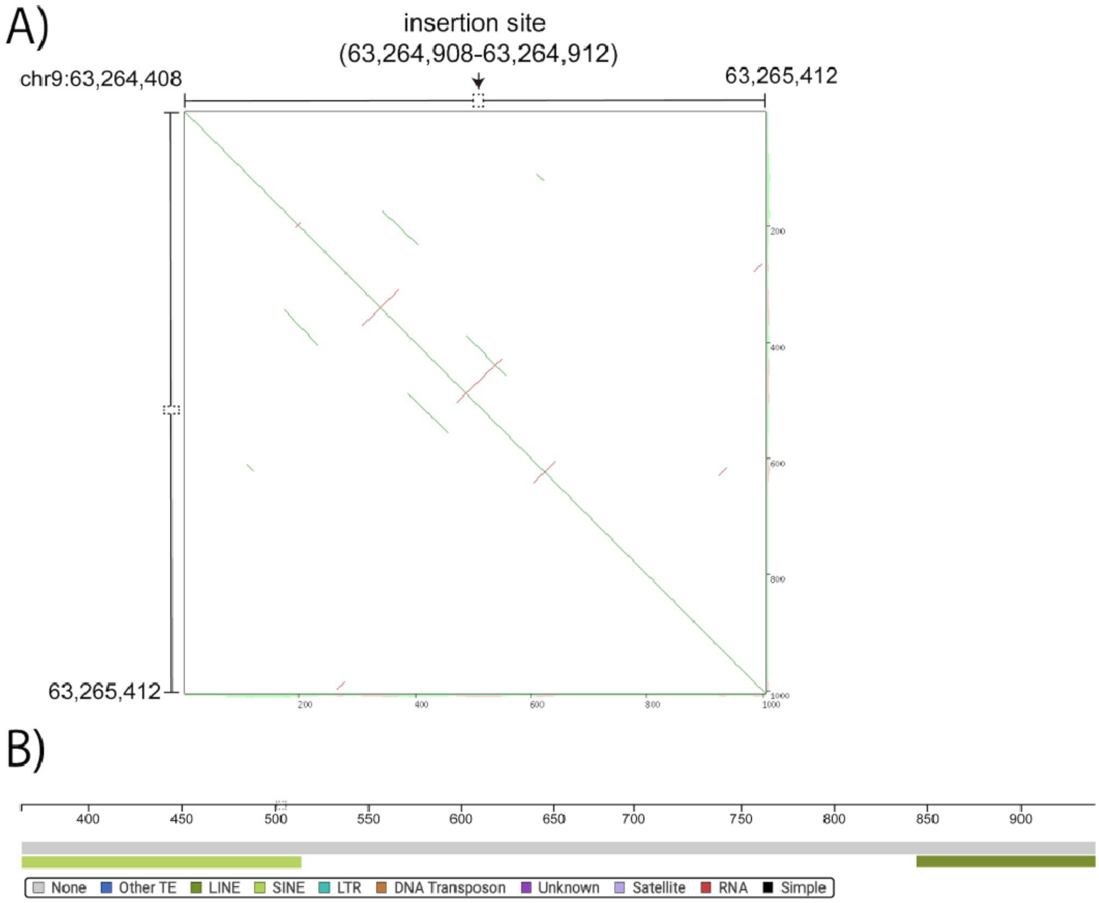

Figure S12. Characterization of ICS#7 flanking sequences.
A) A dot plot indicating DNA sequence similarities, repeat sequences, inversions contained within reference sequence (chr9:63264408-63265412). **B)** Schematic view of repeat elements, predicted by Dfam, located within the ICS flanking region.

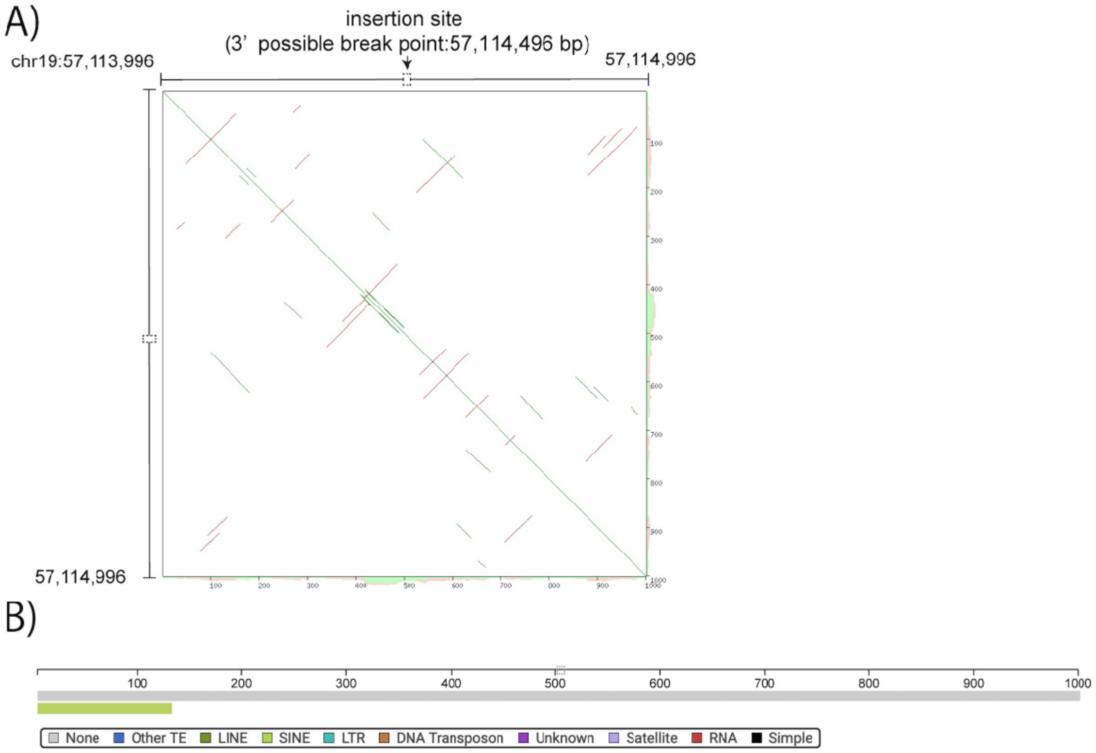

Figure S13. Characterization of ICS#23 flanking sequences.

A) A dot plot indicating DNA sequence similarities, repeat sequences, inversions contained within reference sequence (chr19:57113996-57114996). **B)** Schematic view of repeat elements, predicted by Dfam, located within the ICS flanking region.

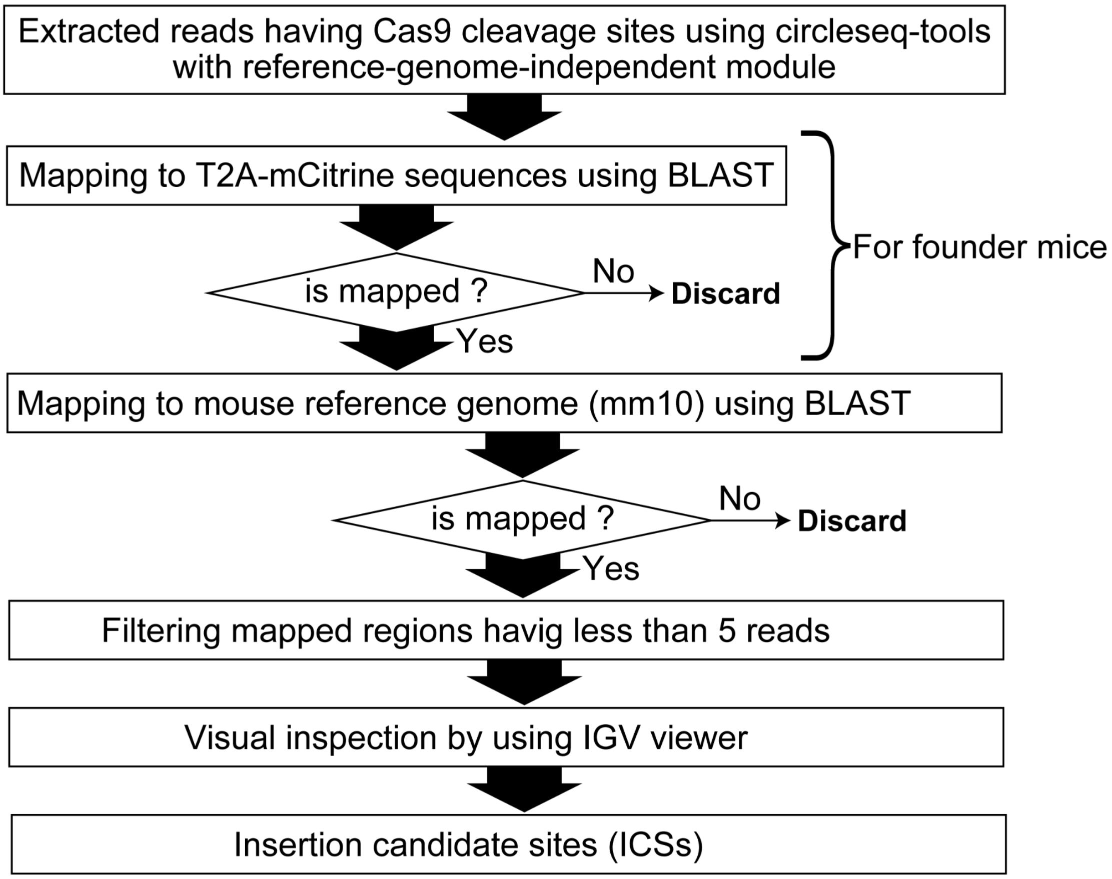

Figure S14. Data analysis flowchart for detection of ICSs.
Flowchart illustrating the procedure of ICS identification.

Table S1. CRISPR target sequences.

gRNA	Sequences	PAM
Mmp9-Cr1	AAGAAGGAGCCCTAGTTCAA	GGG
gRNA-L	CGTAGCCCACGTCGTCCACC	TGG
gRNA-R	ACGAGCTGTACAAGTGAACT	AGG

Table S2. Predicted number of off-target sites for Mmp9-Cr1 gRNA using *in silico* (Cas-OFFinder) and experimentally detected sites by the CRISPR-KRISPR assay.

	Sites with canonical NGG PAM							
	Number of spacer mismatches							
Method	0	1	2	3	4	5	6	Total
Cas-OFFinder	5	7	85	1,363	13,515	113,664	733,648	862,287
CRISPR-KRISPR	1	0	11	109	84	139	214	558
	Sites with non-canonical NGG PAM							
	Number of spacer mismatches							
Method	0	1	2	3	4	5	6	Total
Cas-OFFinder	1	41	1,103	18,033	231,555	755,844	-	1,006,577
CRISPR-KRISPR	0	0	1	19	43	63	118	244

Table S3. Sequences of the oligonucleotides used for OCS detection.

OCSID	Forward sequences	Reverse sequences	product size
OCS0000	CAAATTCTTCTGGCGTGTGAG	GTTTGTGTGTGGTGGTGGTG	187
OCS0001	GAGCCTCTTTGAACTGTGGTG	TCAGACCTTCTCACGATATCCAC	200
OCS0002	AGTTAGCCAGCCTCCATCC	AAGAGCTGGTCGGTAACTCAAA	200
OCS0003	TTGATAATTAACACAACTGACAACAAT	CTGTCTCTAAAACAGAAGCGAAAC	210
OCS0004	AAAGATCCAGACACAAAATATCCAG	TTGGTGTTCTGTACCTTGACAG	189
OCS0005	GGCTCACAGAAGCAGGAAAG	GAGGTGGATGGACAATTCAGA	200
OCS0006	GCCATCTTGACCTGCTAATACAA	TGGCAAGAATGAAGGATGC	198
OCS0007	TTGTGAGTTGTTTAGAGTGCCATTA	CCACCCTTAATGAGAAGCCTG	195
OCS0008	TGCCAGTCCACTTATGTCTCTTC	CCTGAAGGCTCATGGTACTGA	198
OCS0009	CTCGTGAGTTTACATTCATATCTGG	AGTTGCCTAGAGCCATGGAG	205
OCS0010	TGGTGTTCTGTGCACTCTTTGTA	TTAAATACAGGAAAGAAACAGAGAAA	200
OCS0011	GCTTAAGACACCTGTAATAAAATGGC	AAACCCCAAAGGTCTGGAAC	196
OCS0012	AGTTCCCAGGACCTTCTTGC	AGCCGGGCATACACAAAG	195
OCS0013	TTTGCTTTGTTGGTAGGGAAG	ATCAGGTGTCTGAGTTACCTCTTCTAA	196
OCS0014	TCCTCTGTCATGTGTCTGTGTATTC	TGGAGATTTAGCTTAGCTGATGG	200
OCS0015	AAACTAGGAAACATCTTTACCACAAAC	AACATTGATGGAATTGGGTATTTC	202
OCS0016	TGAAGGAGGCATCTGAGACC	TCCTTAGTCACACAGCAAATTCA	198
OCS0017	TGTTGTTCATTTCCCACAATTT	AGAAAAGAAAAGGAGAAAGTGCAA	220
OCS0018	TGGTATGAAACATCCAGGAAAC	TGGTCCAGTCTAATTGATATTTGG	204
OCS0019	CCAGCTACAGGCAGGACAG	TGAGCAGCCATAAAACAAGAA	182
OCS0020	CCCATCTATTTGGGGTTCTATATG	CCCAGGCACAAAACATCC	200
OCS0021	TTCCCGTGTATTGGTGTTCTG	AAGAGGTGAATAGATCAGTCAAAGAAA	220
OCS0022	GATCCCATCCCTCTCCATTC	TGCTGCTGCTTCAGAGATCA	195
OCS0023	CTGACACAAAACATCCTGGAAA	GCTGTTTGGTGTTCTGTATGGT	194
OCS0024	CCAGAGTTTGGATCTCAGCAC	GTCCAGGTACACCTGTGCATAC	200
OCS0025	TAGTGGCTGAAGTTGGAAGAAA	TTTCTACTTAGCTATGCTGCCTTT	206
OCS0026	ATATGCAATGTTTCCCAATTACTG	AAGCTGAAAGTGGCTTACTAAAGAA	208
OCS0027	TTTTGGGGAAGAAAATAACCAA	TGGTAGAAGCAGATGCCAGTC	199
OCS0028	GGCAGTGATGGGACATGG	AGCGATGGGTGGACATAGG	192
OCS0029	TGAAACTCATATACTCCAGTTGAAA	TCTGTATTCTTCTGGTGCCTTTATTA	207
OCS0030	GCCAGAATAAATACTTCTTCCCTTA	GGAGGAAAGTTGTATTGGCTTACA	200
OCS0031	TCCTGATGTATTGTACTTAGTGATTGG	AACTTCCAGACACAAACTATTAAGGAA	211
OCS0032	TCAGGACTTCACACTTGCTCA	TTTGTATTTAGAATTATTTGTCACACC	187
OCS0033	TGGCTATTGAGTTGATTTCCATC	CAGCAATAAAACCCTAACTAAGCAA	207
OCS0034	AAGGGACTTTCTGCTACTTCCA	GCAGCCTAAATAATTGGGATTTG	196
OCS0035	CTGAGCCAATAGATTCACTTAGAGATA	TGCTTCCTTATAGAATCCTGGAC	210
OCS0036	GAACTCCCTAAAACTGACCAAATG	GCATGAGTGATAGCTTATAAAATCTGG	208
OCS0037	AAACTAATTCATGGACTCGTGGT	CTCCACCCAAATTTCTTTTCA	188
OCS0038	TGTGATTCTGTAACCTTTTTGTACTTT	GGACTAAGAAAATGGTAAATCTAAGAA	209
OCS0039	TTGATGTGGTCACTTACTTTGGA	CTTTGGGGACTGTCCTTCC	200
OCS0040	TGTGGAGAGTCATAATTGTTAAACG	TTCCCTCCCTTTCCATGC	197

OCS0041	AGAACAAAAGGAAGCAAATTCA	TGTCCCTTTGCCCTTTAGTG	187
OCS0042	CACCATTTTAAAAAGAGAAAGACAAA	GCCCCCAGCTCTCTTTTAAC	214
OCS0043	GGGAGGGATCACCTAGAAGG	AGTTTAGGTAGGACAGGCTCAATC	190
OCS0044	AAAATGACACAAAACATCTAAAAAGA	TCTTTTCTGGTCCAATCTATGTGA	209
OCS0045	GATCTCATGCCCTCTTCTGG	ATGCTTCCTGTACCCTGATAGAC	206
OCS0046	CATGAACAATTACTCAACCACAGA	TGGTCCTGGAGGATAAGTCA	199
OCS0047	CCCTCTTTAGTAACGGTGCTG	GCAGGGTTAATTGGGACAAA	191
OCS0802	CAACAACAATGCATCCATCC	AGCAATTGTTTGCCTACACTTAGA	184
OCS0803	CAAGTCTTATAAATGATCCAAACCAG	CTTTGAAAATTATAGCGACCAAATC	202
OCS0804	GCAGGGCAGAACCTGAGTAG	GCCTCTCTGGACAGTGGGTA	197

Table S4. Sequences of the oligonucleotides used for ICS detection.

ICS ID	Name	5'->3' sequences
ICS03	ICS-3F	GGCAGCTAACCTTCCTACCTTG
	ICS-3R	ATAGGAGTCAGACGAGAAATACAAC
ICS04	ICS-4F	GCGTTGGCTGTTCCTATTTC
	ICS-4R	CAGGAGGCAGGAGTGGATAC
ICS05	ICS-5F	AAGGCCATCATGAGTCCATAC
	ICS-5R	TCCATGAGGTGGAGTGTGA
ICS06	ICS-6R	ACCAAAGGAAGGATTGAAACTC
	ICS-6F	CACTCCTGGGCAGGATTAAC
ICS07	ICS-7F	ATGTGACTTTGGACTTGGTTTG
	ICS-7R	TAGCGGAGGTCTTAAAGAGTCG
ICS08	ICS-8F	TGCCTCTCTCGTCTGGAAAC
	ICS-8R	CGGGAACAACCATGTCCTAC
ICS09	ICS-9F	GCTGTGTGATGGATGGACTTC
	ICS-9R	AACAGTACAGCAAACAAAGGTGAC
ICS10	ICS-10F	AGTGACATTTGATTGTCTTGTCTTG
	ICS-10R	AGGTTGGCCTGGAGCTTAG
ICS11	ICS-11F	AGCCTTGACACTGCCTTCTG
	ICS-11R	AGAGGCTATAGGGTCTGGATGTG
ICS12	ICS-12F	GGTGACTGAAAAGCTGTTTTGAAC
	ICS-12R	GCCTTCCAAATGAAGCCTGT
ICS13	ICS-13R	CTGTGGAACCACCTCCAAAG
ICS15	ICS-15F	CGTTTGATCCTCCTCAGCAAGAAG
ICS16	ICS-16F	TCAGCCAAGGCCTCTCTTAG
	ICS-16R	CCTGGAAAGCCTGTAGATTGA
ICS17	ICS-17F	TCAACACAGTTAATGCCTCCA
	ICS-17R	GTGGCTGTGCTTCCACACT
ICS18	ICS-18F	CCAAGCAAGCCAGATACAAGA
	ICS-18R	CCCACAAACGTGTGATGTTG
ICS19	ICS-19F	AACAATTTGTGGGCTGGTGA
	ICS-19R	AAAAGTGGCCATGAACTGAAATC
ICS20	ICS-20F	TTCCACAGCTATCTCAGCTTCA
	ICS-20R	TCATAGGAAATCATTTAGCAGAACCT
ICS22	ICS-22F	TGCCGATATCAACCCTAAGC
	ICS-22R	TCTCCCGTTATCCCTGATTG
ICS23	ICS-23R	TGGGGATATGGGTGGGACTTAC
ICS24	ICS-24R	AATGAATCAACAGATCTCCATAAGAAC
ICS25	ICS-25F	CCCATTACTCCCACTCACCTATG
	ICS-25R	GAGCCCCCTTTAAGGAAGAAAG
ICS26	ICS-26F	CAGCACACTCTTCATGCACAAG
	ICS-26R	TTTGAAATTGTGGTGAGAAACAATC
-	M389	TCGCCACCATGGTGAGCAAGGGCGAG

-	M026	GGTGGTGCAGATGAACTTCAG
-	M495	AAGAAGATGGTGCGCTCCTG
-	M245	ATGGTCCTGCTGGAGTTCGT
-	PP210	TGGCGTGAACATCTGAAATC
-	PP211	GAAACAGTCCAACAAGAAAGGAC

Supplementary text: Analysis of the complex mixture of mosaic alleles in the founder mouse 11

The CRISPR-KRISPR analysis revealed a complex mix of mosaic alleles for the founder mouse 11; the results and discussion of this mouse necessitated a separate discussion, which is included here.

The knock-in of T2A-mCitrine cassette into the *Mmp9* locus reported in Quadros et al 2017 [6], had 12 founder mice of which 8 were concluded to contain the insert at the targeted site. This conclusion was based on two junction PCRs (one each at the 5' and 3' junctions). One of the correctly targeted mice (founder 12) died before weaning and thus this mouse could not be included in the CRISPR-KRISPR analysis. Prior to analyzing the genomic DNAs of all the 11 founder mice, we verified their targeted loci using an independent genotyping assay that employed full-length PCR (involving two flanking primers outside the left and right homology arms) (Additional file 1: **Fig. S1C**). This genotyping assay (that was not performed previously) revealed that the founder 11 (one of the 8 correctly targeted mouse), contained an imprecise insertion as it produced much bigger amplicon than expected. The genotyping image using the two junctional PCR performed in Quadros et al. (Additional file 1: **Fig. S1B**) [6], and the flanking primer PCR done in this study (Additional file 1: **Fig. S1C**) are shown side-by-side in the supplementary figure to indicate the differences in the bands for the founder 11 (Additional file 1: **Figs. S1B** and **S1C**).

Further characterization one of the ICSs (ICS#15) revealed a complex mix of mosaic alleles, all of those existed in the founder 11. Here we provide the description of at least four different mosaic alleles we could decipher from CRISPR-KRISPR analyses.

1) As described in the main manuscript, we identified imprecise insertion (ICS#10/#15) in the founder 11 by PCR using ICS15F (designed specifically for this purpose) and M389 (**Fig. 4C**) primer set. We call ICS#10/#15 as allele 1 (figure on the right, see also **Fig. 4** in the main manuscript).

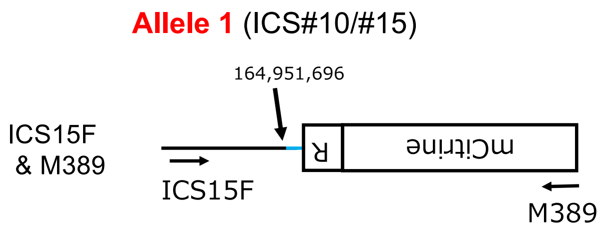

2,3) Based on ICS#15, we used ICS15F primer along with PP211 primer that was supposed to amplify the ICS#10/#15 insertion. This PCR produced a band corresponding to wild-type allele (3.6kb), two prominent bands and several much bigger sized bands. We isolated the two prominent bands, named them as alleles 2 and 3 (see figure below) that were cloned and sequenced. We found that the allele 2 had correct insertion junction for the right arm, but it had about ~1.5kb deletion including the PP210 primer binding site and PAM sequence for gRNA-L, meaning that the left arm was truncated. As for the allele 3, it did not contain T2A-mCitrine cassette but had ~1.4kb deletion including the on target insertion site.

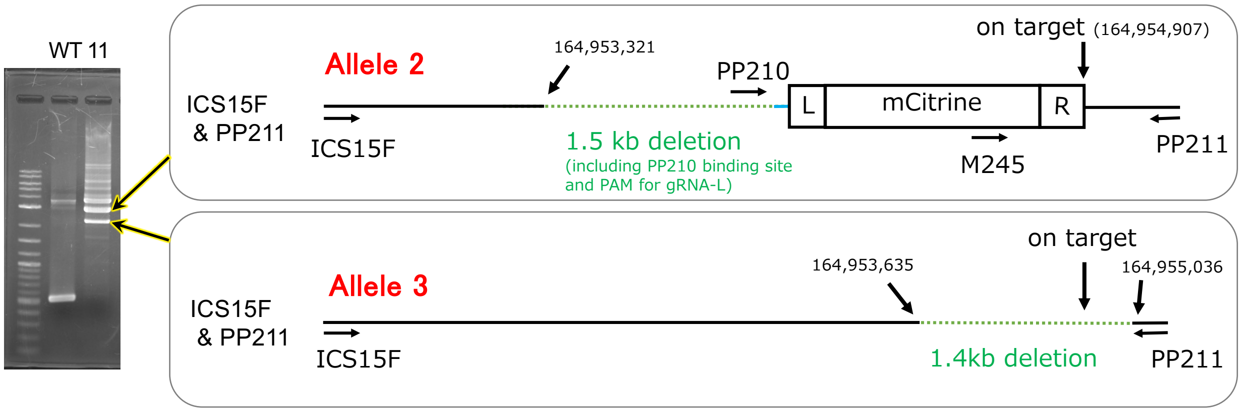

4) Our attempt to clone the larger amplicon of PP210 and PP211 (Additional file 1: **Fig. S1C**) was unsuccessful and thus we performed direct sequencing of this fragment after gel purification. This experiment revealed partial sequence containing 5' junction using PP210 primer (figure on the right), "*" indicates the undetermined region in the 3' end of the insert.

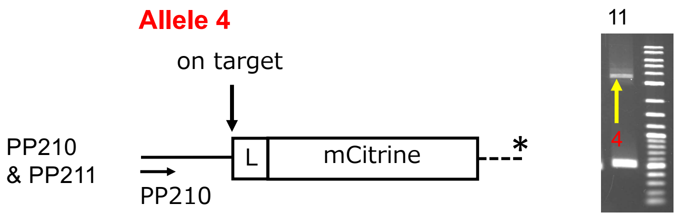

From these analyses, we speculate that two separate alleles contributed to the positive junctional PCR genotyping assays reported in our previous report [6]: the alleles 4 and 2 contributed to positive bands for the 5' and 3' junction PCRs respectively.

Conclusions from further analysis of founder 11 mosaic alleles:

CRISPR-KRISPR assay can be helpful in ruling out imprecise insertions among the founders that were thought to be correctly inserted based on the junctional genotyping PCRs. Among the 6 (out of 11) founder mice that contain correctly targeted alleles, we found that one mouse (founder 1) had allele with incorrect insertion at its 3' junction (ICS#4) and two (founders 6 and 7) also contained additional RIs (ICS#3/#14 for founder 6, ICS#5/#9 for founder 7) (**Fig. 4D**, **Table 1**) in addition to correctly targeted alleles. Although correctly targeted alleles, among mixtures of mosaic alleles and/or RIs elsewhere, could be segregated by breeding (in a situation like this where many founders contained correctly targeted alleles), a researcher can focus only on the founders (that do not contain other unwanted alleles or confounding mosaic alleles) by screening all positive founders by CRISPR-KRISPR prior to choosing bets founders for breeding to establish F1 generation mice. Because of practical considerations, many

researchers expand only one or two founder mice for further experiments and discontinue the other founder lines. In this particular example of knock-in mouse project, CRISPR-KRISPR assay helped us in ruling out one (or two) of the presumed correctly targeted founders as not good ones to choose for next generation breeding. Performing CRISPR-KRISPR assay in a scenario like this can help exclude some founders for further breeding.
