# Supplementary material for: CRISPR-KRISPR: a method to identify on-target and random insertion of donor DNAs and their characterization in knock-in mice
Source: Genome Biol. 2022 Oct 25;23:228. doi: 10.1186/s13059-022-02779-8 (PMC9594901; doi:10.1186/s13059-022-02779-8)
Supplement: Supplementary file 2 — Additional file 2. Review history. [file 13059_2022_2779_MOESM2_ESM.docx]

Review History

**First round of review**

**Reviewer 1**

**Were you able to assess all statistics in the manuscript, including the appropriateness of statistical tests used?** No.

**Were you able to directly test the methods?** No.

**Comments to author:**

Major comments
1. How does this method compare to other established approaches for assessing random integrations (RIs)? In this study, authors used CRISPR-KRISPR analysis or PCR-based approaches to identify RIs. However, other established methods such as quantifying the copy number of mCitrine integration by ddPCR, southern blotting, ligation adapter-mediated PCR (LAM-PCR) etc. can straightforwardly evaluate the RIs.

2. Is the distance of homology-arm targeting gRNAs critical for the method? In figure 3, gRNA-L is targeted for left arm and gRNA-R targeted for an overlap of cassette donor and right arm, does the position and distance of these gRNAs important for this assay?

3. Could the CRISPR-KRISPR method detect partial random-integration (RI) events? gRNAs were designed at the junctions of the cassettes (left and right arms) but there is a possibility of partial RI events occurring that are not detectable. How could this assay help in detecting partial RIs?

4. How comprehensively is CRISPR-KRISPR detecting unintended random insertion (RI)? Whole genome sequencing of one or two founder animals might help answer this question. Also, authors discuss about the use of Oxford Nanopore for dsDNA donor cassettes which will have high error rates for long range sequencing, how will authors rationalize it?

5. What is the basis to conclude that CRISPR-KRISPR can be broadly used for off-targets and random insertions in knock-in targets? This study performed using a single gRNA Mmp9-Cr1 that showed no detectable off-target activity at 50 off-target candidate sites (OCS) analyzed in mouse early embryos with modified CIRCLE-seq (802 sites). Random Insertion candidate sites (ICS) 23 by CRISPR-KRISPR have no overlap with OCS sites. Would identifying ICS by CRISPR-KRISPR at the different targets with similar cassette show greater generalizability of this assay?

6. Regards to discussion section (line 308): The authors claim that CRISPR-KRISPR is a 'unified method' for identifying both off-target activity and random insertion events. However, it seems that CRISPR-KRISPR would need to be performed separately using on-target gRNAs and gRNAs targeted to the homology arms. The authors should clarify this point in the discussion.

Minor comments
1. What supports the conclusion that 'no off-target mutations were introduced in mouse embryos? This conclusion should be more accurately and rigorously stated as 'No off-target mutations were detected at X candidate sites above a limit of detection of approximately 0.1% in edited mouse embryos.
2. How comparable is the CRISPR-KRISPR modification of CIRCLE-seq to the original protocol? Authors have modified genomic DNA shearing protocol, have they adjusted any key steps and how comparable these two protocols are?
3. What is the percentage of on-target editing at Mmp9-Cr1 locus with Cas9 and knock-in efficiency with T2A-mCitrine cassette donor?
Most of the data presented for on-target editing is evaluated by number of CHANGE-seq identified reads and in vitro cleavage assay. It is important to evaluate the Cas9 mediated editing at the Mmp9 target loci and knock-in efficiency with T2A-mCitrine cassette.
4. What type of Cas9 used?
5. Many typos require correction, here are few examples.
Typo at line 31 in abstract, CRISPR-KRISPR approach
Typo errors at lines 90, 114 for CIRCLE-seq

**Reviewer 2**

**Were you able to assess all statistics in the manuscript, including the appropriateness of statistical tests used?** Yes: The major area for statistical analysis was in the number of sequencing reads for each OCS, and the minimum number was shown and exceeded for each site.

**Were you able to directly test the methods?** No.

**Comments to author:**

The authors have made modifications to the established Circle-seq protocol that will more accurately identify insertions at off-target sites as well as concurrently identifying sites of random insertions of the DNA sequence. This is especially useful for identifying candidate lines of modified mice for further study when multiple lines are available. Overall, this is a very detailed paper with careful analysis of possible artifacts and appropriate controls. There are a couple of minor points that I would make as a reviewer. To the editors, it will be essential to make certain that the figures are clear and large enough to see the detail required. To the authors, it would be very helpful to have an initial figure (I would suggest as Fig. 1--perhaps Fig 3. should be Fig.1??) that schematically details both the CircleSeq method and the CRISPR/KRISPR method, showing how they differ and what the end results would be. This would then set the stage for showing your work and results that the reader can then appreciate to a greater degree.

In the methods, it is important to note, please, that DNA was isolated both from wt and from mutant mice. Other than this, the methods are quite clear. This technique could prove incredibly useful for those doing CRISPR modification in cells provided they can obtain single clones for analysis.

Finally, in the discussion, you may wish to note that there was a publication discussing artifacts that could be found during genotyping of DNA that was sheared by enzymatic fragmentation (Gregory T, et al: NAR GEnomics and Bioinformatics 2020 Vol2(4). Perhaps your unexpected insertions in/near the transgene could be explained by this? Just something to consider.

**Authors Response**

**Point-by-point responses to the reviewers’ comments:**

Reviewer #1:

Major comments

1. How does this method compare to other established approaches for assessing random integrations (RIs)? In this study, authors used CRISPR-KRISPR analysis or PCR-based approaches to identify RIs. However, other established methods such as quantifying the copy number of mCitrine integration by ddPCR, southern blotting, ligation adapter-mediated PCR (LAM-PCR) etc. can straightforwardly evaluate the RIs.

***Our primary goal of modifying the Circle-seq method was to make it useful for identifying CRISPR off target cleavages but then as we were developing the method, we realized it could also be used for RI detection. Among the three methods the reviewer has suggested, southern blotting (SB) and ddPCR cannot identify RIs accurately because they lack a sequencing step to know the location/identity of RI. Although SB was a primary method used for differentiating correct targeting and RI in the ES cell-based targeting, it is a laborious method (involving screening of several probes to find the ones that work best, and also it requires optimization of hybridization condition with radioactivity that is decreasingly utilized in basic research due to regulatory factors and expense, waste, etc). Therefore, it is unattractive for many researchers to use it. This is particularly the case for analyzing mice generated using the CRISPR approach in which mosaicism adds another whole layer of complexity. In regards to ddPCR, this method is typically used for copy number identification, not for identifying RI per se. ddPCR assay can be indicative of RIs (based on higher copy numbers than expected) but it cannot identify the locations of RIs, again because it does not involve sequencing step.***

***Another alternative method to ddPCR (to identify copy numbers) is qPCR. We performed qPCR of the mice; data included in the revised manuscript (Fig. S1D). In this assay, we used an eGFP knock-in mouse as a control. We designed qPCR primers in the regions of eGFP and mCitrine sequences that are identical. We set the numeric number 2 based on the values of homozygous eGFP mouse sample (in this case, heterozygous mouse sample will have a value of 1). In this assay, ideally, the three founders (1,6 and 7) that contained RIs in addition to the on-target insertion should show a value higher than 2. However, only founder 7 showed higher than 2. The founders 1 and 6 showed less than 2 even though they contained RI because they had only one correct insertion on target site (heterozygous) and therefore the value of 2 in these founder mice was because 1 count came from on target insertion and another 1 count came from the RI. Therefore, just by qPCR it is not possible to conclude of a founder mouse has RI or not, at least in founder mice (but qPCR will be applicable in next generation F1 mice). Another major problem in using a non-NGS based approaches like (SB, ddPCR or qPCR) is mosaicism, which will interfere with accurate interpretations of results. Even though SB, qPCR or ddPCR can be used for knowing additional insertions in F1 offspring (where mosaicism is not a concern), but still the assays can only indicate presence of RI but they cannot reveal the identity of RI sites. Lastly, regarding LM-PCR approach, it can identify the locations of RIs when the method is used in combination with sequencing. However, one of the advantages of CRISPR-KRISPR over LM-PCR is that the same experimental procedure can be used for identification of OT and RI in CRISPR-KRISPR.***

2. Is the distance of homology-arm targeting gRNAs critical for the method? In figure 3, gRNA-L is targeted for left arm and gRNA-R targeted for an overlap of cassette donor and right arm, does the position and distance of these gRNAs important for this assay?

***The position and distance of gRNA from the cassette and arm boundaries depend directly on the NGS read lengths (used for detecting ICS). If the NGS read lengths can be up to a few kilobases long, the location of guides used for CRISPR-KRISPR analysis can be any anywhere within the insert cassette. We used a Miseq v2 with a read length of 150 bp*2 as the next-generation sequencer for ICS and RI detection. Our assumption was that the sequence of one read can be read up to 150 bp, and therefore it should contain the boundary between the arm and RI sites (the other 150 bp read contains the T2A-mCitrine sequence), and the obtained genomic sequence must be long enough to determine which region on the genome it is. With the above rational, we designed the gRNAs inside the arm or at the boundary between the arm and the cassette (depending on the availability of PAM sequences). Note that the knock-in model designs used as test case in this study contain very short homology arms (~60 bases). If Miseq v2 with read length of 250 bp*2 is used, gRNAs may be designed more inside the cassette rather than in the arm region, as the total read length obtained will be longer. As stated above, if the read length can be gained more than a few kb by using a long-read sequencer, it does not matter in which regions of the insert the gRNAs are designed. In summary, the location and distance of gRNAs are considered dependent on NGS read length.***

3. Could the CRISPR-KRISPR method detect partial random-integration (RI) events? gRNAs were designed at the junctions of the cassettes (left and right arms) but there is a possibility of partial RI events occurring that are not detectable. How could this assay help in detecting partial RIs?

***We think that our method can detect partial insertions including the one with truncated arm as long as the inserted sequence contains the gRNA binding site in it. However, exhaustive detection of partial insertions is a limitation if guides to only certain regions of the insert are used. A possible solution to this would be to design gRNAs at certain intervals throughout the knock-in cassette and perform the same experiment to detect partial random insertions. However, this would require the use of many gRNAs, which can increase the cost of performing the assay. These points were not discussed in the manuscript and are included in the revised manuscript. and are copied below for your convenience***

***It is possible that partial insertions of truncated fragment can occur in knock-in experiments. Note that we used two guides, one each in the homology arm regions, and as expected, the RIs identified all contained the guide binding sites. The CRISPR-KRISPR method can possibly be used for identifying partial insertions of different regions of the insertion cassette if gRNAs binding to different regions throughout the cassette are included in the assay.***

4. How comprehensively is CRISPR-KRISPR detecting unintended random insertion (RI)? Whole genome sequencing of one or two founder animals might help answer this question. Also, authors discuss about the use of Oxford Nanopore for dsDNA donor cassettes which will have high error rates for long range sequencing, how will authors rationalize it?

***It is not known at this point how comprehensively CRISPR-KRISPR can detect random insertions (RIs). We agree with the reviewer that WGS approach may help answer this question. However, one of the limitations of WGS is that it may not provide enough information to draw clear conclusions by analyzing founder mice, which may have only a few random insertion sites in the genome at most. In addition, if a mutation with a low allele frequency, such as a mosaic RI, is present in the founder mice, it may be difficult to identify it without deep sequencing, and such a comprehensive analysis may require considerable cost. .***

***As for Oxford Nanopore sequencing, it is indeed difficult to analyze at single nucleotide resolution due to many sequencing errors. However, we do not think that this is a big problem for detecting flanking regions of RIs, which is the overall goal of our method to detect RI. In other words, longer read lengths are expected to detect RI flanking regions of the genome, even if sequencing has some errors. In addition, the chemistry of the Oxford Nanopore has recently been improved to enable more accurate sequencing (the R10.4 flow cell is 99% accurate), which should solve the problem of higher error rates. In regard to this, a recent preprint article demonstrates the value of technical improvement in LRS methods for detecting transgene insertion sites https://www.biorxiv.org/content/10.1101/2022.01.05.475144v1.***

5. What is the basis to conclude that CRISPR-KRISPR can be broadly used for off-targets and random insertions in knock-in targets? This study performed using a single gRNA Mmp9-Cr1 that showed no detectable off-target activity at 50 off-target candidate sites (OCS) analyzed in mouse early embryos with modified CIRCLE-seq (802 sites). Random Insertion candidate sites (ICS) 23 by CRISPR-KRISPR have no overlap with OCS sites. Would identifying ICS by CRISPR-KRISPR at the different targets with similar cassette show greater generalizability of this assay?

***We realized that the terminology ‘broad’ is not a correct choice here. We intended to say that this method can detect both OT and RI unlike those that can only detect either OT or RI. We apologize for creating confusion with the incorrect use of the term ‘broad’. We have modified the text accordingly to indicate the point that ‘our method can detect both OT for the knock-in guide and RI of donor DNA just by using the guide RNA for knock-in and another gRNA designed on donor DNA in the CRISPR-KRISPR experimental design’. As for the OT detection, which was the first objective of this method development, we could not find any. This was expected because several papers show that CRISPR-generated mice rarely have OTs. As stated above (in response to comment 1), we realized that our method can also detect RI. The conclusion that potential OT sies and ICS do not overlap suggests that RI can occur independently of OT cleavage. Generating models with a similar cassette at different targets for to examine its generalizability may not be feasible. However, considering that we thoroughly analyzed the full set of 11 founder mice to demonstrate the application of our method, we believe it can be applied to other loci as well for different insertion cassettes.***

6. Regards to discussion section (line 308): The authors claim that CRISPR-KRISPR is a 'unified method' for identifying both off-target activity and random insertion events. However, it seems that CRISPR-KRISPR would need to be performed separately using on-target gRNAs and gRNAs targeted to the homology arms. The authors should clarify this point in the discussion.

***We agree that separate reactions need to be performed for different guide RNAs (one for OT and one for RI). However, the experimental procedure for each set of guides is same. We modified the sentence in the manuscript to clarify this confusion. The sentence has been revised as follows.***

***The CRISPR-KRISPR method helps identify, or rule out, both OT and RI as a unified method by using several gRNAs in the experiment: the knock-in gRNA for OT detection and gRNA(s) binding to the donor DNA for RI detection, respectively.***

Minor comments

1. What supports the conclusion that 'no off-target mutations were introduced in mouse embryos? This conclusion should be more accurately and rigorously stated as 'No off-target mutations were detected at X candidate sites above a limit of detection of approximately 0.1% in edited mouse embryos.

***Since CIRCLE-seq is characterized by the fact that sites where DSBs are likely to occur (typically on-target sites) are at the top of the read counts, we considered that it would be sufficient to validate the Top 47 OCSs out of 802 OCSs and 3 OCSs uniquely detected in silico and performed deep amplicon sequencing. We revised the results and discussion section as follows.***

***We conclude that Mmp9-Cr1 gRNA did not cause OT mutations at 50 OCSs above a limit of detection of approximately 0.1 %.***

***The OT mutations in the knock-in mice were below the detection limit and were insignificant as reported previously in the literature***

2. How comparable is the CRISPR-KRISPR modification of CIRCLE-seq to the original protocol? Authors have modified genomic DNA shearing protocol, have they adjusted any key steps and how comparable these two protocols are?

***In the CRISPR-KRISPR method, we changed the fragmentation step (from physical fragmentation to enzymatic fragmentation), and the steps after circularized DNA preparation were same as in CIRCLE-seq (we added a schematic diagram as Figure 1 to describe the differences between the CIRCLE-seq and CRISPR-KRISPR methods). This modification greatly reduced the amount of genomic DNA required in our hands, and we believe that the fragmentation step changes had little effect on the results, since CRISPR-KRISPR also showed a tendency that the sites where DSBs are likely to occur, a characteristic of CIRCLE-seq, were ranked higher in the number of reads.***

3. What is the percentage of on-target editing at Mmp9-Cr1 locus with Cas9 and knock-in efficiency with T2A-mCitrine cassette donor?

Most of the data presented for on-target editing is evaluated by number of CHANGE-seq identified reads and in vitro cleavage assay. It is important to evaluate the Cas9 mediated editing at the Mmp9 target loci and knock-in efficiency with T2A-mCitrine cassette.

***The CRISPR-KRISPR results show that on-target reads were obtained in 2,088 of 36,858 sites, which represents the highest percentage (approx. 5.7%). The in vitro cleavage efficiency at the on-target sites was very high (94.7%), and the in vivo editing efficiency using this gRNA was observed in 11 of the 11 mice examined in this study (100% including all KI and indel-only edited mice). Of these, the correct targeted alleles were observed in the 7 of 11 mice 63.6(%), indicating that the in vivo editing efficiency by Mmp9-Cr1 is also very high.***

4. What type of Cas9 used?

***The Alt-R® S.p. Cas9 Nuclease (Integrated DNA Technologies) was used. We added this information in the materials and methods.***

5. Many typos require correction, here are few examples.

Typo at line 31 in abstract, CRISPR-KRISPR approach

Typo errors at lines 90, 114 for CIRCLE-seq

***We thank the reviewer for identifying the typos. We corrected them.***

Reviewer #2:

The authors have made modifications to the established Circle-seq protocol that will more accurately identify insertions at off-target sites as well as concurrently identifying sites of random insertions of the DNA sequence. This is especially useful for identifying candidate lines of modified mice for further study when multiple lines are available. Overall, this is a very detailed paper with careful analysis of possible artifacts and appropriate controls. There are a couple of minor points that I would make as a reviewer. To the editors, it will be essential to make certain that the figures are clear and large enough to see the detail required. To the authors, it would be very helpful to have an initial figure (I would suggest as Fig. 1--perhaps Fig 3. should be Fig.1??) that schematically details both the CircleSeq method and the CRISPR/KRISPR method, showing how they differ and what the end results would be. This would then set the stage for showing your work and results that the reader can then appreciate to a greater degree.

In the methods, it is important to note, please, that DNA was isolated both from wt and from mutant mice. Other than this, the methods are quite clear. This technique could prove incredibly useful for those doing CRISPR modification in cells provided they can obtain single clones for analysis.

Finally, in the discussion, you may wish to note that there was a publication discussing artifacts that could be found during genotyping of DNA that was sheared by enzymatic fragmentation (Gregory T, et al: NAR GEnomics and Bioinformatics 2020 Vol2(4). Perhaps your unexpected insertions in/near the transgene could be explained by this? Just something to consider.

***We thank the reviewer for the comments. We modified the manuscript accordingly. Specifically, we added a schematic diagram (as Figure 1) to describe the differences between the Circle-Seq and the CRISPR-KRISPR methods. We also noted in the METHODS SECTION that genomic DNA was extracted from wild-type and founder mice and used as follows.***

***C57BL/6J mice and eleven founder mice obtained by Easi-CRISPR using repair template containing T2A-mCitrine cassette were used for CRISPR-KRISPR experiments [6]. Genomic DNAs were extracted from these mouse tails using the DNeasy Blood & Tissue Kit (Qiagen).***

***We thank the reviewer for the useful information about the artifact by Gregory et al. In the Discussion section, we mentioned the advantage of the enzymatic fragmentation step in reducing the amount of DNA, but we also mentioned the possibility of artifacts specifically associated with this enzymatic fragmentation step in the discussion section, and we cited the paper. In our analysis, we did not examine such artifacts in detail, and it is possible that they may be present to some extent in the reads. However, if our interpretation is correct, the artifact comes from "regions of nearby DNA from opposite strands (by Gregory et al)," which would map to regions close to the RI site. In this case, it would be necessary to take into consideration the direction of knock-in. In our method, we designed the experiment to detect RI in either direction (using both F and R primers: Fig. 4B), so we do not think we missed many RIs.***

**Second round of review**

**Reviewer 1**

The authors have appropriately addressed the reviewer's comments and the revised version of this manuscript is satisfactory.
